# Supplementary material for: Comprehensive molecular characterization of collecting duct carcinoma for therapeutic vulnerability
Source: EMBO Mol Med. 2024 Aug 9;16(9):2132–45. doi: 10.1038/s44321-024-00102-5 (PMC11393068; doi:10.1038/s44321-024-00102-5)
Supplement: Supplementary file 1 — Appendix [file 44321_2024_102_MOESM1_ESM.pdf]

Appendix Information

Comprehensive Molecular Characterization of Collecting Duct Carcinoma for Therapeutic Vulnerability

Peiyong Guan[1,#], Jianfeng Chen[2,#], Chengqiang Mo[3,#], Tomoya Fukawa [4,#], Chao Zhang[5], Xiuyu Cai[2], Mei Li[2,6], Jing Han Hong[7], Jason Yongsheng Chan[8], Cedric Chuan Young Ng[9], Jing Yi Lee[9], Suet Far Wong[9], Wei Liu[9], Xian Zeng[2], Peili Wang[2], Rong Xiao[2], Vikneswari Rajasegaran[9], Swe Swe Myint[9], Abner Ming Sun Lim[9], Joe Poh Sheng Yeong[10,11], Puay Hoon Tan[11,12,13], Choon Kiat Ong[14], Tao Xu[15], Yiqing Du[15], Fan Bai[16], Xin Yao[5,*], Bin Tean Teh[1,7,9,17,*], Jing Tan[2,9,18*]

Affiliations

[1] Genome Institute of Singapore (GIS), Agency for Science, Technology and Research (A*STAR), Singapore

[2] State Key Laboratory of Oncology in South China, Guangdong Provincial Clinical Research Center for Cancer, Collaborative Innovation Center for Cancer Medicine, Sun Yat-sen University Cancer Center, Guangzhou, P. R. China.

[3] Department of Urology, The First Affiliated Hospital of Sun Yat-sen University, PR China

[4] Department of Urology, Tokushima University Graduate School of Biomedical Sciences, Tokushima, Japan

[5] Department of Genitourinary Oncology, Tianjin Medical University Cancer Institute and Hospital, National Clinical Research Center of Cancer, Tianjin Key Laboratory of Cancer Prevention and Therapy, Tianjin's Clinical Research Center for Cancer, Tianjin, P. R. China

[6] Department of Pathology, Sun Yat-sen University Cancer Center, Guangzhou, PR China.

[7] Cancer and Stem Cell Biology Programme, Duke-NUS Medical School, Singapore

[8] Division of Medical Oncology, National Cancer Centre Singapore, Singapore

[9] Laboratory of Cancer Epigenome, Division of Medical Sciences, National Cancer Centre Singapore, Singapore

[10] Institute of Molecular and Cell Biology (IMCB), Agency of Science, Technology and Research (A*STAR), Singapore, Singapore

[11] Department of Anatomical Pathology, Singapore General Hospital, Singapore, Singapore

[12] Division of Pathology, Singapore General Hospital, Singapore, Singapore

[13] Luma Medical Centre, Singapore

[14] Lymphoma Genomic Translational Research Laboratory, National Cancer Centre Singapore, Singapore

[15] Department of Urology, Peking University People's Hospital, Beijing 100044, China

[16] Biomedical Pioneering Innovation Center (BIOPIC), Beijing Advanced Innovation Center for Genomics (ICG), School of Life Sciences, Peking University, Beijing 100871, China

[17] SingHealth/Duke-NUS Institute of Precision Medicine, National Heart Centre Singapore, Singapore

[18] Hainan Academy of Medical Science, Hainan Medical University, Haikou, PR China

#These authors contributed equally.

***Corresponding authors**

Appendix Information Table of Contents

Appendix Figure S1 2
Appendix Figure S2 6
Appendix Figure S3 7
Appendix Table S1 8
Appendix Table S2 13

Appendix Figure S1

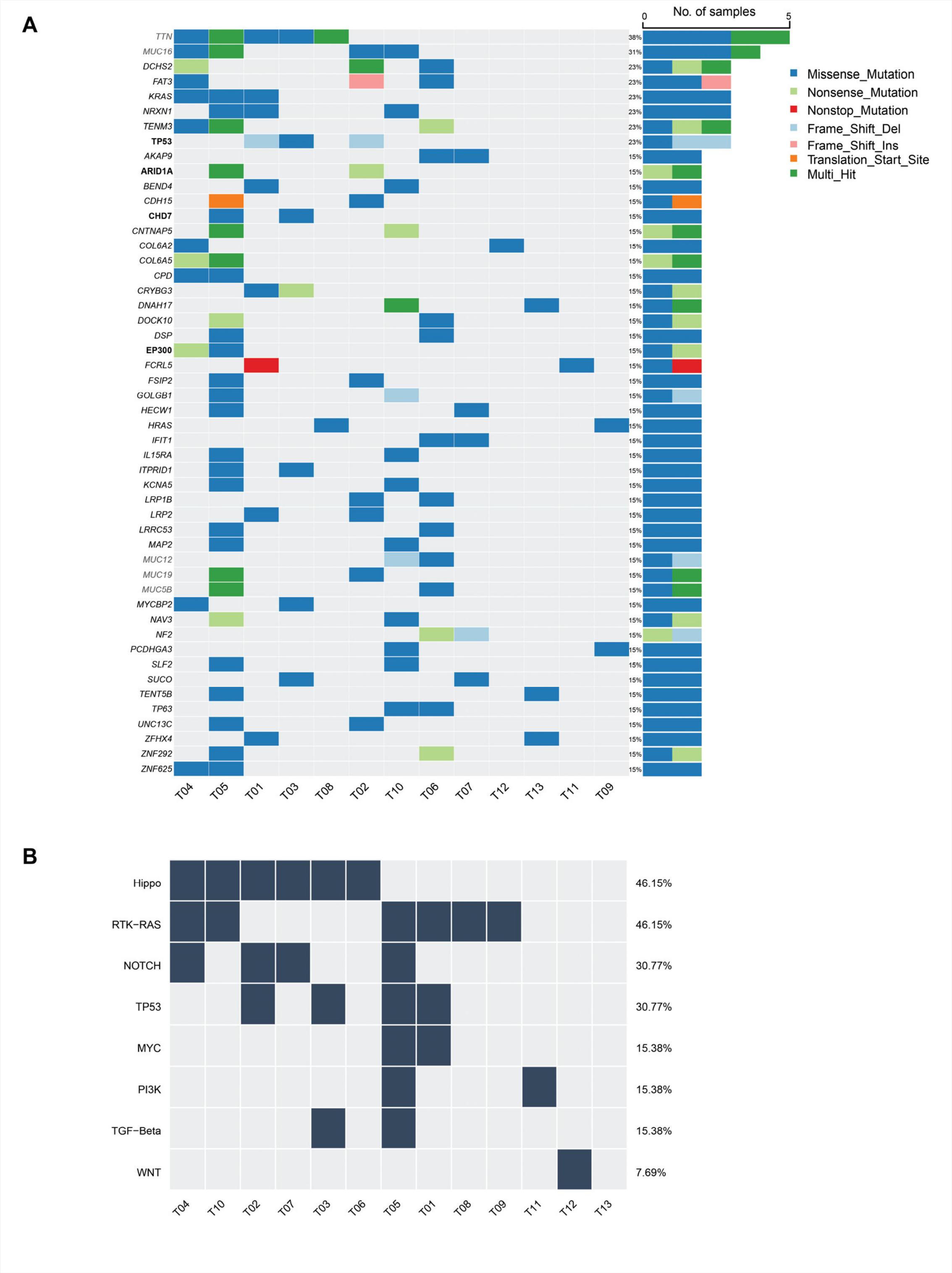

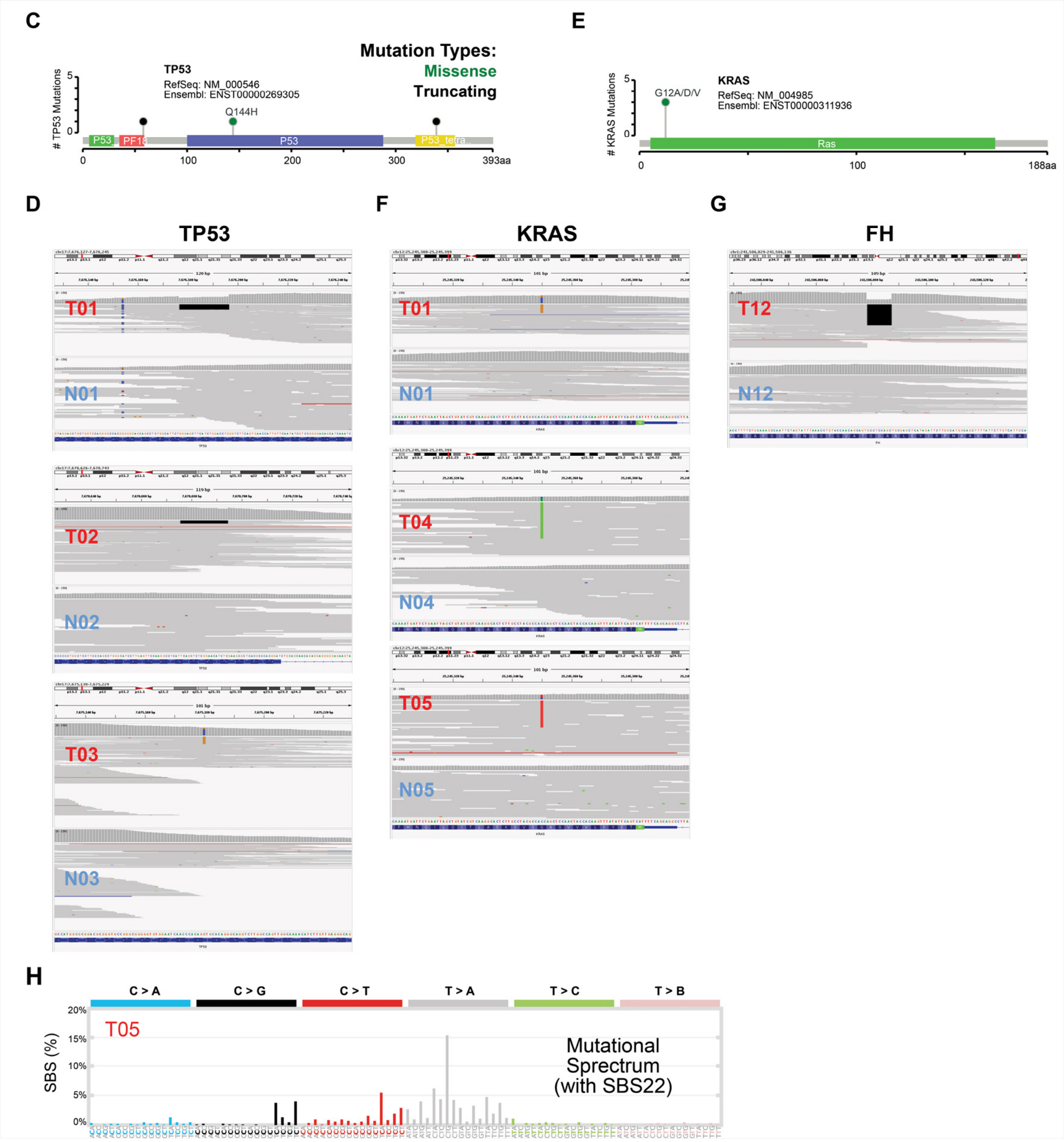

C
Mutation Types:
Missense
Truncating
TP53
RefSeq: NM_000546
Ensembl: ENST00000269305
TP53 Mutations
Q144H
P53
P53
0
100
200
300
393aa
E
KRAS
RefSeq: NM_004985
Ensembl: ENST00000311936
KRAS Mutations
G12A/D/V
Ras
0
100
188aa
D
TP53
T01
N01
T02
N02
T03
N03
F
KRAS
T01
N01
T04
N04
T05
N05
G
FH
T12
N12
H
C > A
C > G
C > T
T > A
T > C
T > B
SBS (%)
20%
15%
10%
5%
0%
T05
Mutational Sprectrum (with SBS22)

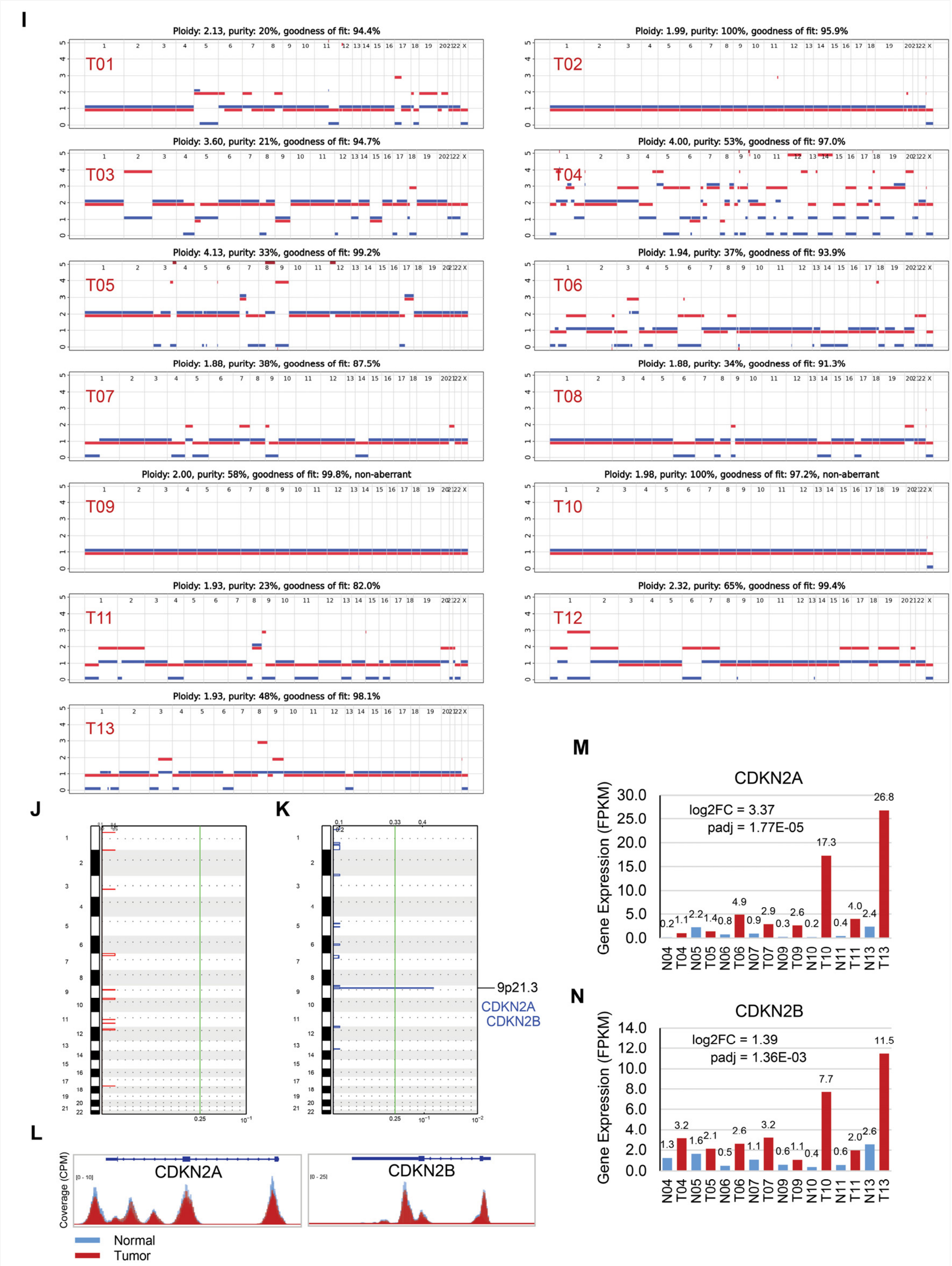

Appendix Figure S1. Genomic landscape of collecting duct carcinoma.

(A) Recurrent mutations. Epigenetic genes are highlighted in bold. Frequently mutated genes (grey colored) across many cancer types, such as TTN, MUC16, MUC12, MUC19, MUC5B, are presented for completeness. (B) Mutated oncogenic pathways in the samples. (C) Lollipop plot for TP53 mutations. (D) IGV (Integrative Genomics Viewer) plots for sequencing reads mapped at the mutations of TP53 locus. (E) Lollipop plot for KRAS hotspot mutations. (F) IGV plots for sequencing

reads mapped at the mutations of KRAS locus. (G) IGV plots for sequencing reads mapped at the mutations FH gene locus. (H) Mutational spectrum of T05, which has aristolochic acid mutational signature (SBS22). (I) Allele-specific copy number analysis of tumors (ASCAT) profiles. (J) No significant amplification predicated by GISTIC2.0. (K) GISTIC2.0 predicated focal deletion at 9p21.3, where genes CDKN2A and CDKN2B are located. (L) Overlayed tracks showing normalized sequencing coverage at CDKN2A and CDKN2B loci, where the coverages in the normal samples are relatively higher. CPM: counts per million (Group Autoscale in IGV, Integrative Genomics Viewer). (M) CDKN2A expression levels in the normal (cyan) and tumor (red) samples. FPKM: Fragments Per Kilobase of transcript per Million mapped reads. (N) CDKN2A expression levels in the normal (cyan) and tumor (red) samples.

Appendix Figure S2

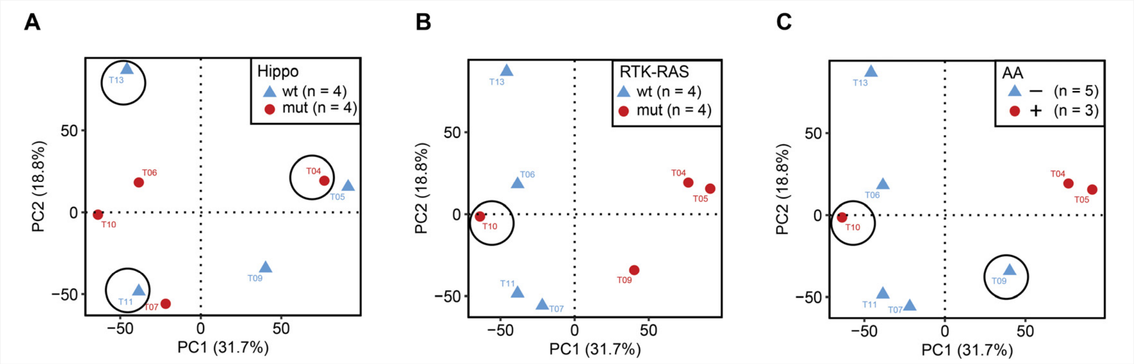

Appendix Figure S2. Genomic mutation and associated transcriptomic alteration inform precision therapy.

(A) Principal component analysis (PCA) of samples used in Hippo pathway analysis. (B) PCA of samples used in RTK-RAS pathway analysis. (C) PCA of samples used in AA positive (+) and negative (-) analysis. Circled samples were excluded from comparisons, because they did not cluster transcriptomically with majority of the samples sharing the same mutated pathway. This allowed us to study relatively more homogeneous groups of samples.

Appendix Figure S3

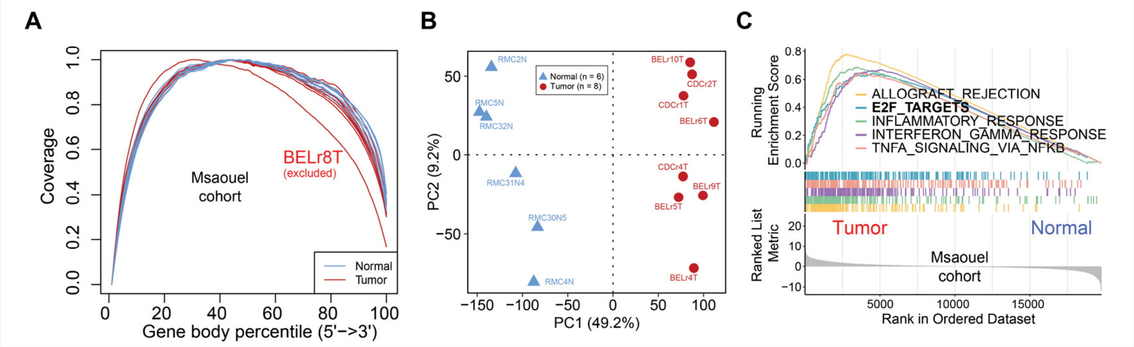

Appendix Figure S3. Transcriptome of collecting duct carcinoma.

(A) Reads distribution along the gene body (Msaouel cohort). BELr8T has slight bias to 5’ of the gene body and was excluded from further analysis. (B) Principal component analysis (PCA) of Msaouel cohort (n = 6 for normal; n = 8 for tumor). (C) Gene set enrichment analysis (GSEA) of Msaouel cohort, top five enriched Hallmark pathways are shown.

Appendix Table S1. Clinical data.

Patient ID	Gender	Age	Op Date	Date of Death	Days Survived	Metastasis/ Recurrence	Tissue Sample	Germline Tissue Available	WES	RNAseq	Pathological Diagnosis
CDC1	male	51	n.a.	n.a.	n.a.	metastasis	fresh	No	Yes	Yes	Postoperative distant (spinal) metastasis from the left renal collecting duct.<br>• Primary tumor<br>*Immunohistochemistry*: CK(+), Vim(+), CK(H, partially +), CK(L+), CAM5.2(+), EMA(+), CK19(+), CK18(+), CK8 (+), CD15 (few cells +), , Ki67 (about 2%+), CD34 (blood vessels +), D2-40 (lymphatic vessels +), CD10(-), Villin(-), CK7(-).<br>*Diagnosis*: (Left kidney) Microscopic histomorphological changes combined with immunohistochemistry results are consistent with renal Bellini collecting duct carcinoma, and the cancer tissue invades the perirenal adipose tissue.<br>• Metastatic tumor<br>*Immunohistochemistry*: Heterocyst, CK(+), PAX-8(+), CK(HMW)(-), TTF-1(-), CDX-2(-), CK7(-)<br>*Diagnosis*: Based on the HE morphology, immunohistochemistry and medical history, the lesion is consistent with metastasis of renal cell carcinoma (thoracic spine). Please refer to the

Patient ID	Gender	Age	Op Date	Date of Death	Days Survived	Metastasis/ Recurrence	Tissue Sample	Germline Tissue Available	WES	RNAseq	Pathological Diagnosis
											pathology report of the primary tumor for tumor classification.
P01	male	68	n.a.	n.a.	n.a.	n.a.	fresh frozen	Yes	Yes	No	p63, cytokeratin (CAM5.2, 32βE12), positive.
P02	male	73	n.a.	n.a.	n.a.	n.a.	fresh frozen	Yes	Yes	No	Right nephrectomy specimen. Immunohistochemical staining results: CK (+), Vimentin (weak +), CD10 (+), CA-IX (weak +), CK7 (-), P504S (-), CD117 (-), GATA-3 (weak +), PAX-2 (-), PAX-8 (-), 34 BE12 (+), p63 (+), p40 (+), TFE-3 (+), Ki-67 (30% +). (Note: The tumor is immunohistochemically TFE3 positive, and if necessary, molecular biomonitoring is excluded except for Xp11.2 translocation renal cell carcinoma).
P03	male	42	n.a.	n.a.	n.a.	n.a.	fresh frozen	Yes	Yes	No	cytokeratin (CAM5.2, 19), Vimentin, positive.

Patient ID	Gender	Age	Op Date	Date of Death	Days Survived	Metastasis/ Recurrence	Tissue Sample	Germline Tissue Available	WES	RNAseq	Pathological Diagnosis
P04	male	58	2012-03-19	2012-06-13	86	metastasis	fresh frozen	Yes	Yes	Yes	1. (Left) Poorly differentiated renal cancer, considered collecting duct cancer, has invaded the renal capsule, ureteral stump (-), perirenal fat sac (-), and 2/2 of the cancer metastasis can be seen in the left renal hilum lymph node; 2. (Left) adrenal gland (-). Immunohistochemistry: CK high, CK low, EMA and CK7 are all (+), BCL-2, vimentin part (+), ki-67>50%, CK20 (-).
P05	female	82	2018-07-16	2018-12-01	138	no metastasis	fresh frozen	Yes	Yes	Yes	High-grade renal cell carcinoma of the right kidney, consistent with collecting duct carcinoma, accompanied by massive necrosis, suspicious vascular invasion, involvement of renal cortex and renal sinus fat, and no cancer metastasis.
P06	female	72	2014-06-10	2014-12-01	174	metastasis	fresh frozen	Yes	Yes	Yes	Left renal cell carcinoma, tending to collecting duct carcinoma, metastatic carcinoma can be seen in renal hilar lymph nodes 1/1.
P07	female	62	2012-06-10	2014-06-01	721	metastasis	fresh frozen	Yes	Yes	Yes	Left renal adenocarcinoma, collecting duct cancer is considered, metastasis cannot be ruled out, please combine clinical observation, ureteral stump (-), fat cyst (-).

Patient ID	Gender	Age	Op Date	Date of Death	Days Survived	Metastasis/ Recurrence	Tissue Sample	Germline Tissue Available	WES	RNAseq	Pathological Diagnosis
P08	male	80	n.a.	n.a.	n.a.	n.a.	fresh frozen	Yes	Yes	No	Right nephrectomy specimen. Immunohistochemical staining results: CK7 (+), CK20 (-), CD10 (focal +), Vimentin (scattered tumor cells +), p63 (+), CK5 / 6 (+), CK19 (+), 34βE12 (+), CD15 (-), villin (-), PAS (-), AB (+), PAX8 (+).
P09	female	54	2018-04-03	2019-09-01	516	brain metastasis	fresh frozen	Yes	Yes	Yes	Left renal collecting duct carcinoma, involving renal sinus fat, renal pelvis (+)
P10	male	50	2020-06-30	Alive, last follow up 2020-11-18	141	lymph node metastasis	fresh frozen	Yes	Yes	Yes	The right kidney combined with immunohistochemistry is consistent with a malignant tumor of collecting duct origin, tending to collecting duct carcinoma; small foci of vascular infiltration can be seen in the renal sinus fibrous tissue.
P11	female	45	2010-06-26	2011-09-16	447	metastasis	fresh frozen	Yes	Yes	Yes	(Left) Renal collecting duct carcinoma, grade III, invades the renal capsule, renal pelvis stump (-), renal fat cyst (+).
P12	female	40	n.a.	n.a.	n.a.	lymph node metastasis	fresh frozen	Yes	Yes	No	Renal cell carcinoma, 10.0 cm in dimension, Fuhrman nuclear grade 4. Tumor extends to the collecting system, renal sinus and perirenal fat.

Patient ID	Gender	Age	Op Date	Date of Death	Days Survived	Metastasis/ Recurrence	Tissue Sample	Germline Tissue Available	WES	RNAseq	Pathological Diagnosis
P13	male	69	2015-09-22	2017-09-14	723	recurrence	fresh frozen	Yes	Yes	Yes	(Left kidney) High-grade renal cell carcinoma, combined with immunohistochemistry results, is considered to be collecting duct cancer, ureteral stump (-), perirenal fat sac (-), renal portal vasculature (-), left renal hilar soft tissue (-), a little adrenal tissue can be seen; Immunohistochemistry: CK7 (-), Vimentin (-), CD117 (-), P504S (+), CD10 (focal +), TFE-3 (-), CA9 (-) , broad-spectrum (+), CK8/18 (+), P63 (-), 34βE12 (-), CK19 (+), PAX-8 (partial +)

Appendix Table S2. Whole exome sequencing quality checking.

Sample ID	Patient ID	Coverage (X)	chrY coverage (x)	Gender	Insert Size (bp)
CDC1(PDX)	CDC1	81.21	49.57	XY	259
N01	P01	120.87	21.35	XY	151
T01	P01	126.82	16.16	XY	145
N02	P02	97.4	65.44	XY	164
T02	P02	117.95	58.08	XY	171
N03	P03	128.21	21.04	XY	153
T03	P03	141.36	20.47	XY	152
N04	P04	92.87	61.25	XY	200
T04	P04	88.25	42.25	XY	201
N05	P05	85.27	0.82	XX	306
T05	P05	78	0.72	XX	319
N06	P06	110.31	0.85	XX	304
T06	P06	112.75	0.77	XX	309
N07	P07	81.43	1.09	XX	214
T07	P07	79.6	1.18	XX	194
N08	P08	120.15	88.36	XY	163
T08	P08	117.13	68.96	XY	160
N09	P09	79.9	0.64	XX	301
T09	P09	78.9	0.59	XX	304
N10	P10	119.64	92.64	XY	275
T10	P10	119.85	72.86	XY	266
N11	P11	88.33	1.19	XX	203
T11	P11	88.45	1.08	XX	215
T12	P12	144.04	0.1	XX	141
N13	P13	94.58	72.52	XY	330
T13	P13	81.31	58.83	XY	319
