# Supplementary figures and images for: Comprehensive molecular characterization of collecting duct carcinoma for therapeutic vulnerability
Source: EMBO Mol Med. 2024 Aug 9;16(9):2132–45. doi: 10.1038/s44321-024-00102-5 (PMC11393068; doi:10.1038/s44321-024-00102-5)

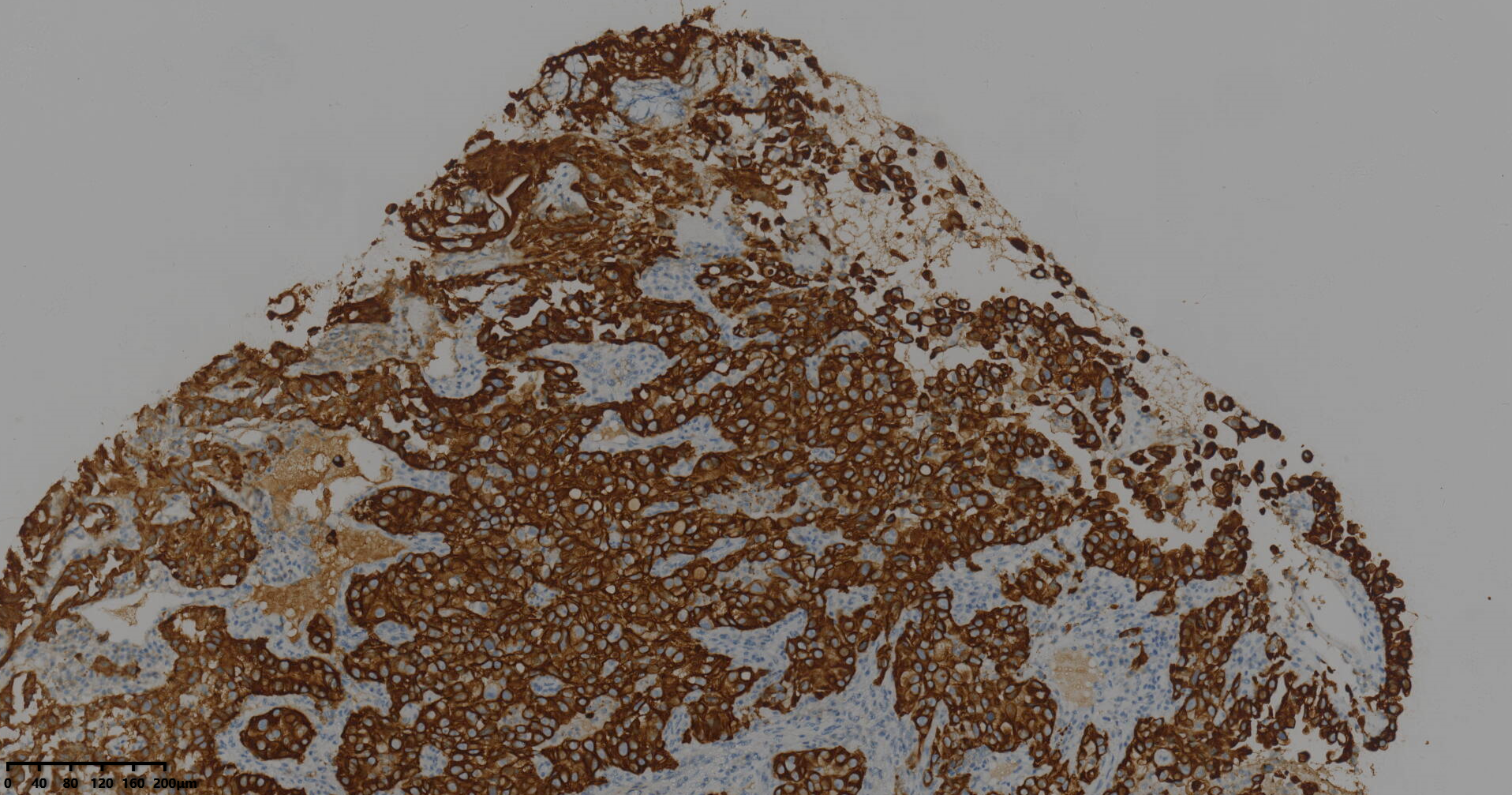

Supplement: Supplementary file 7 — Source data Fig. 4 [file 44321_2024_102_MOESM7_ESM.zip › Figure 4/4B/Patient-CK19.tiff]

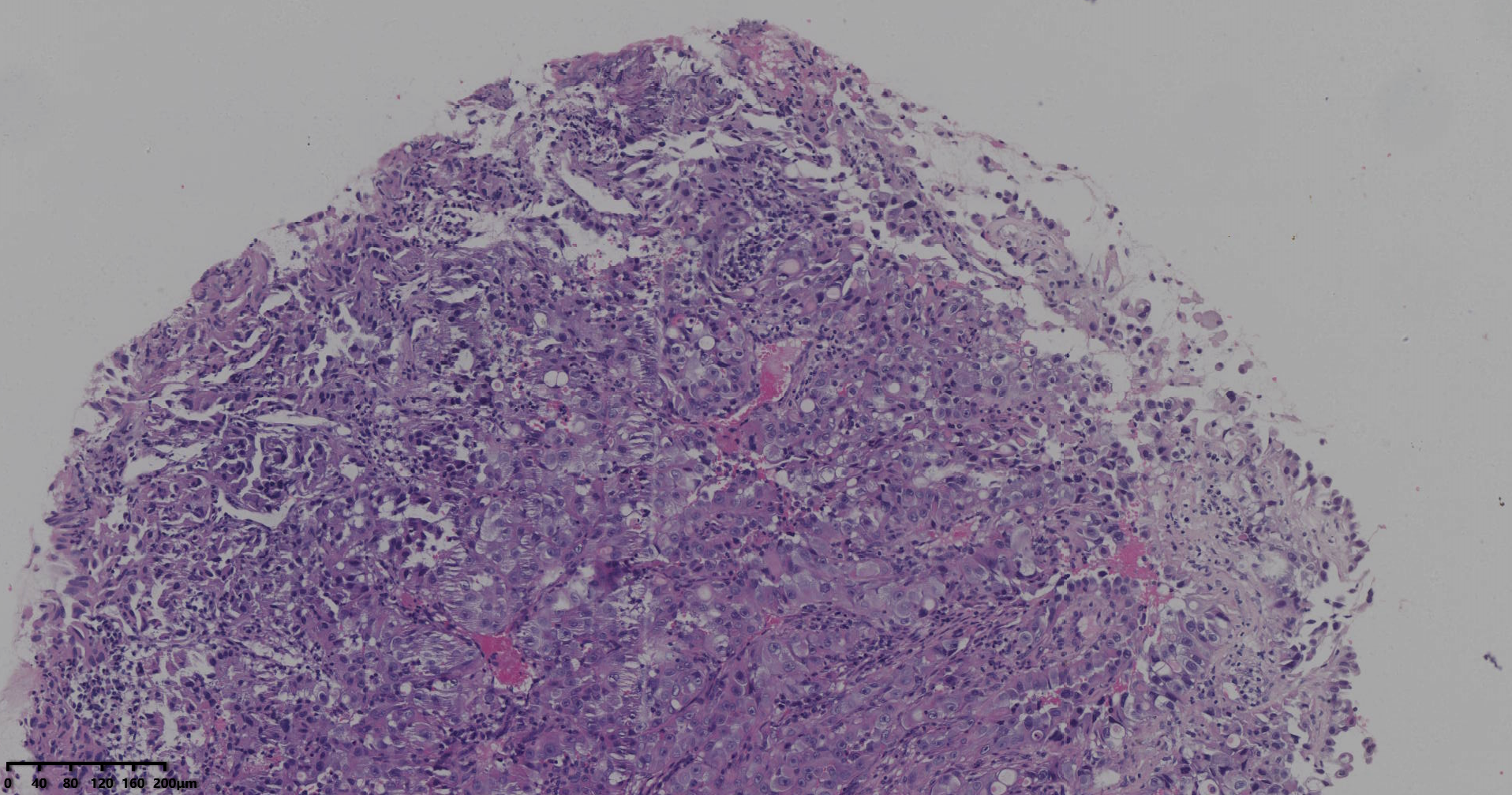

Supplement: Supplementary file 7 — Source data Fig. 4 [file 44321_2024_102_MOESM7_ESM.zip › Figure 4/4B/Patient-HE.tiff]

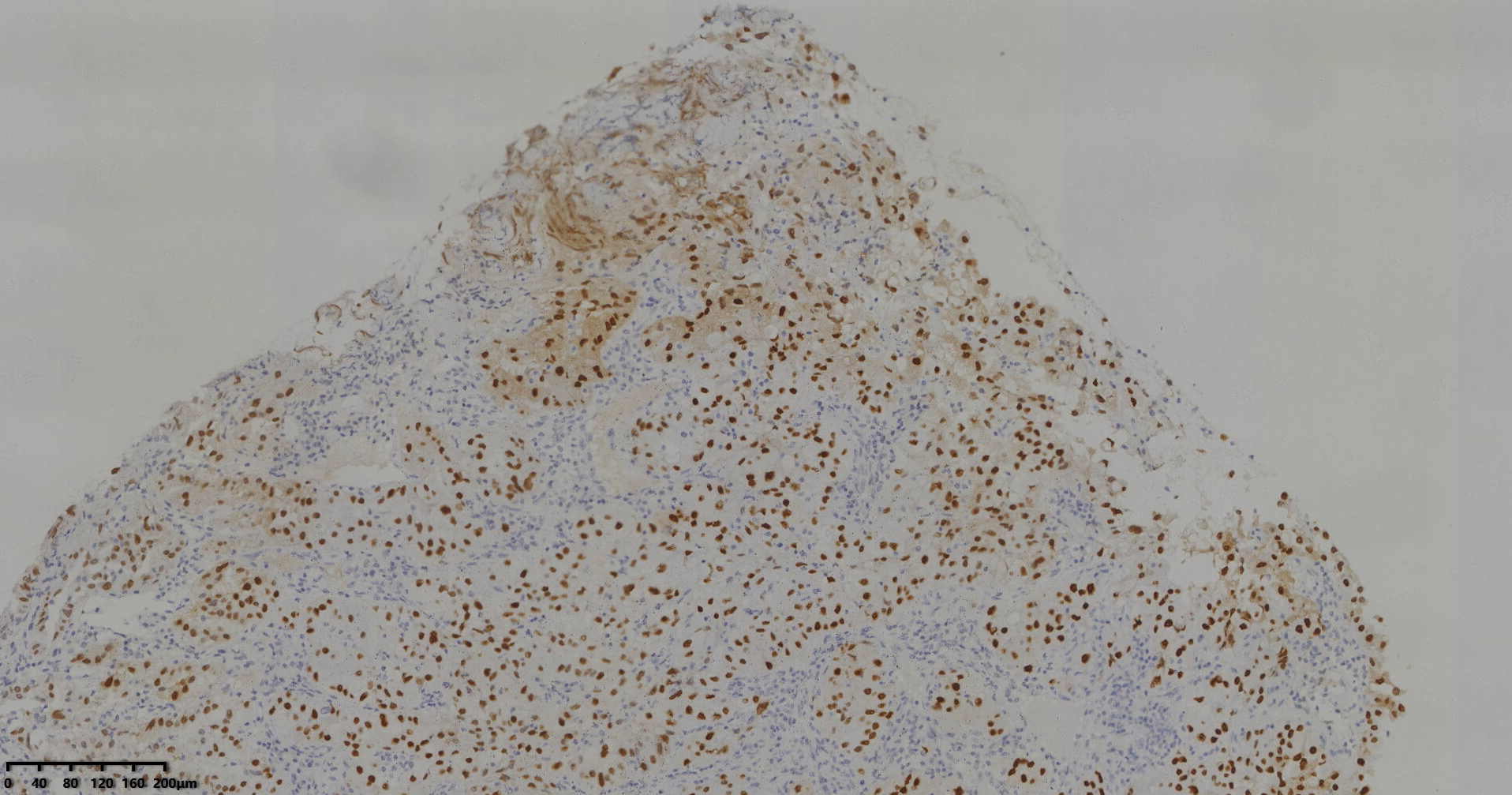

Supplement: Supplementary file 7 — Source data Fig. 4 [file 44321_2024_102_MOESM7_ESM.zip › Figure 4/4B/Patient-PAX8.tiff]

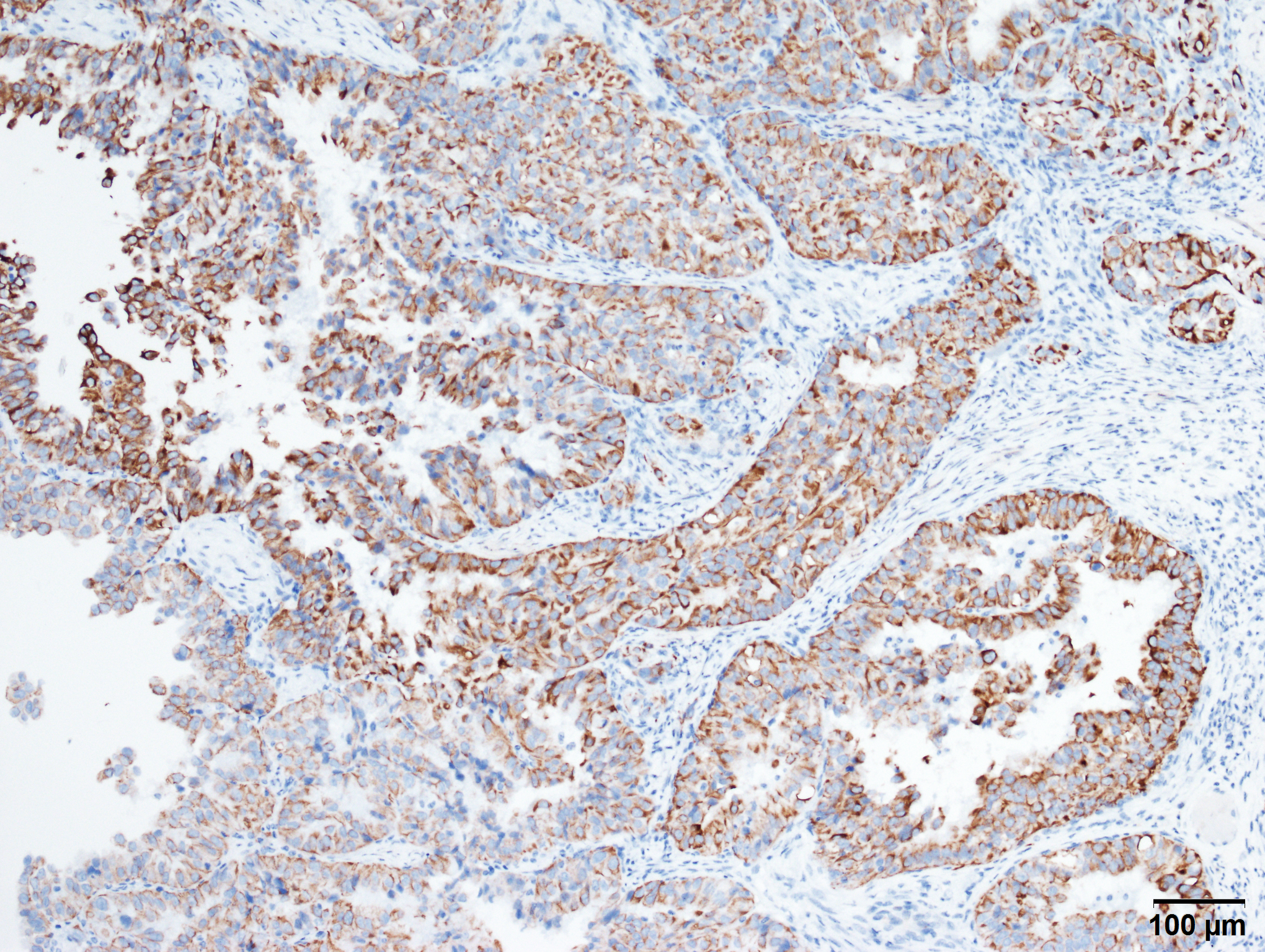

Supplement: Supplementary file 7 — Source data Fig. 4 [file 44321_2024_102_MOESM7_ESM.zip › Figure 4/4B/PDX-CK19-100.tif]

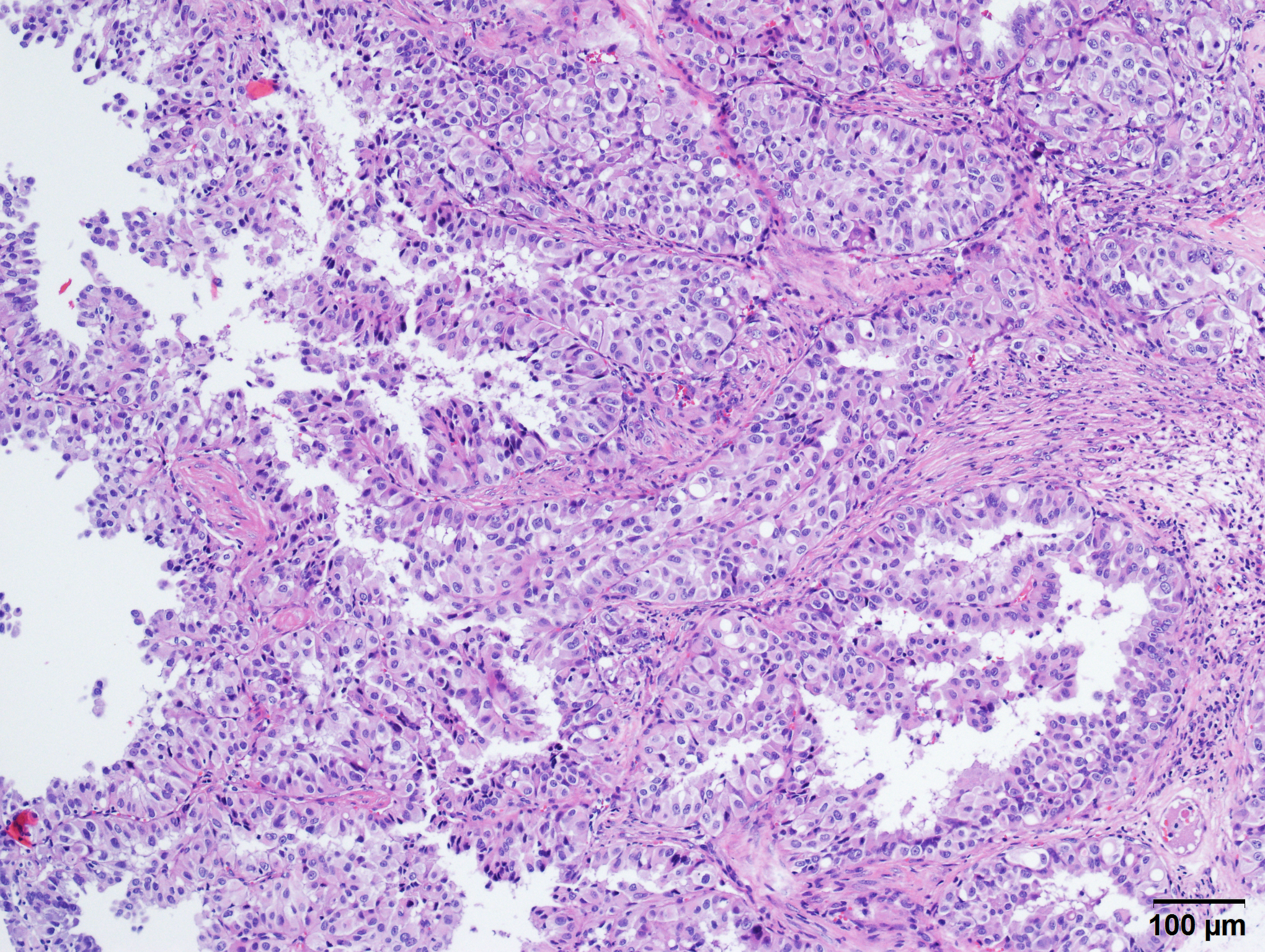

Supplement: Supplementary file 7 — Source data Fig. 4 [file 44321_2024_102_MOESM7_ESM.zip › Figure 4/4B/PDX-HE-100.tif]

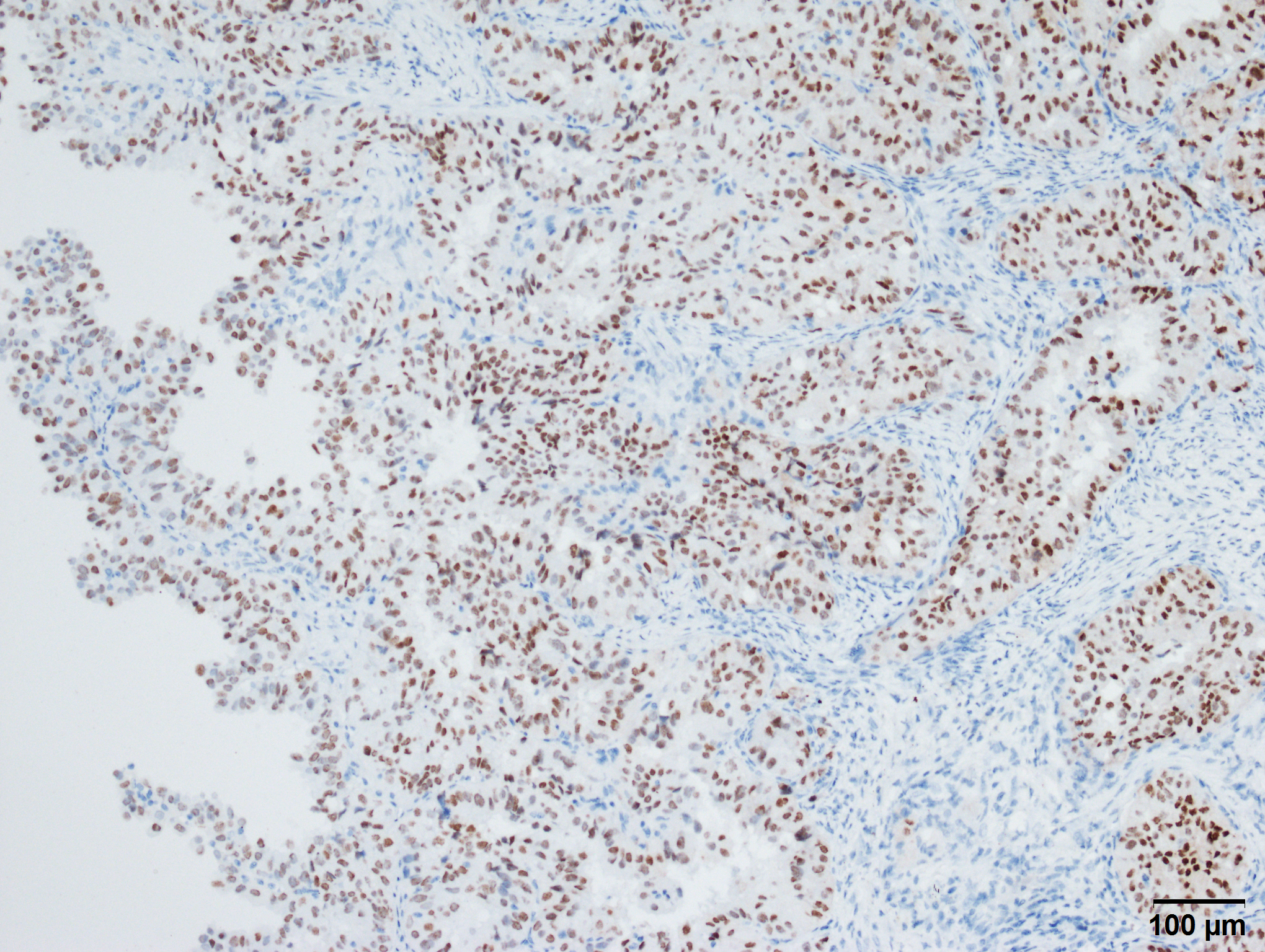

Supplement: Supplementary file 7 — Source data Fig. 4 [file 44321_2024_102_MOESM7_ESM.zip › Figure 4/4B/PDX-PAX8-100.tif]

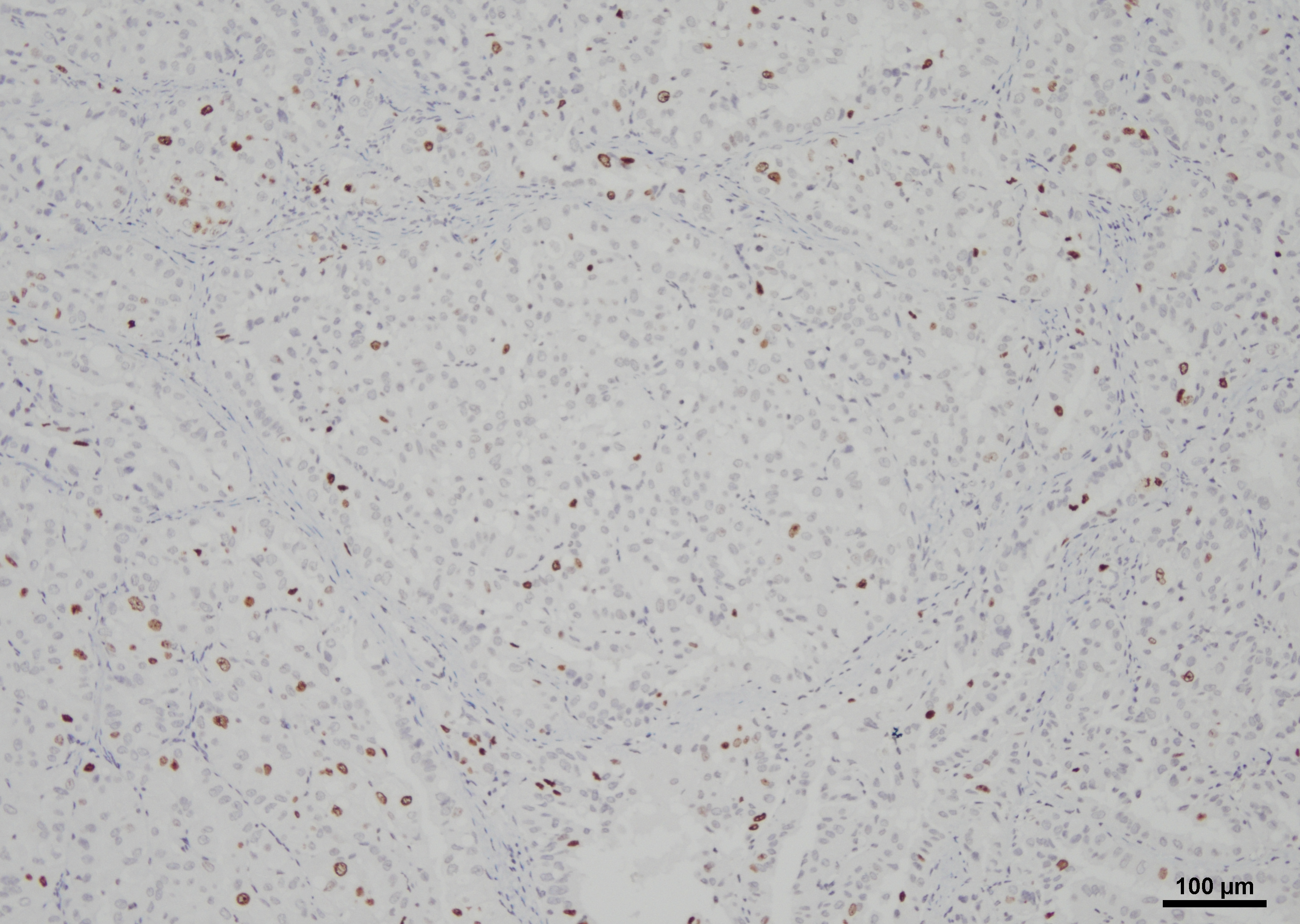

Supplement: Supplementary file 8 — Source data Fig. 5 [file 44321_2024_102_MOESM8_ESM.zip › Figure 5/5D/LDC-Ki67-10X.tif]

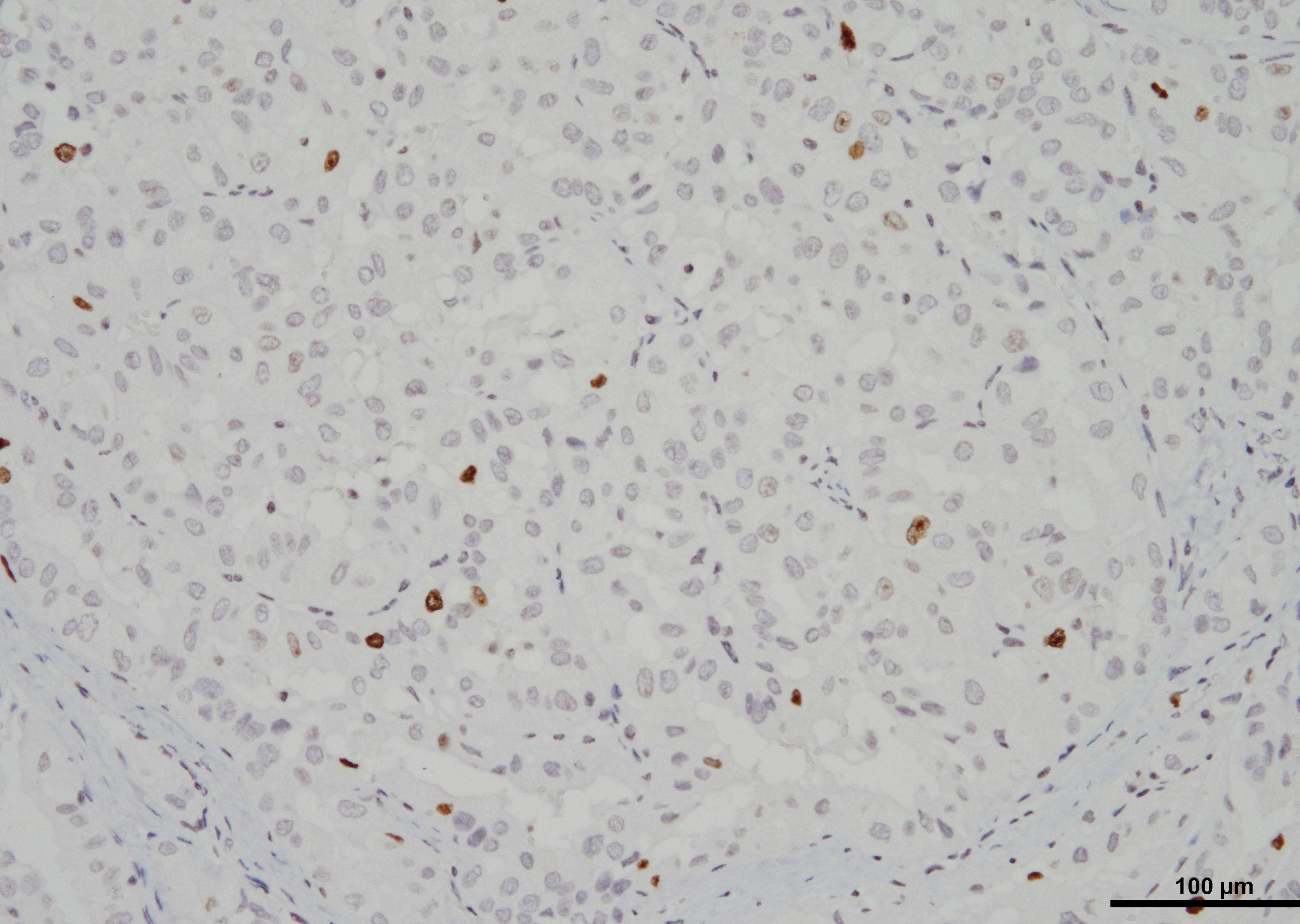

Supplement: Supplementary file 8 — Source data Fig. 5 [file 44321_2024_102_MOESM8_ESM.zip › Figure 5/5D/LDC-Ki67-20X.tif]

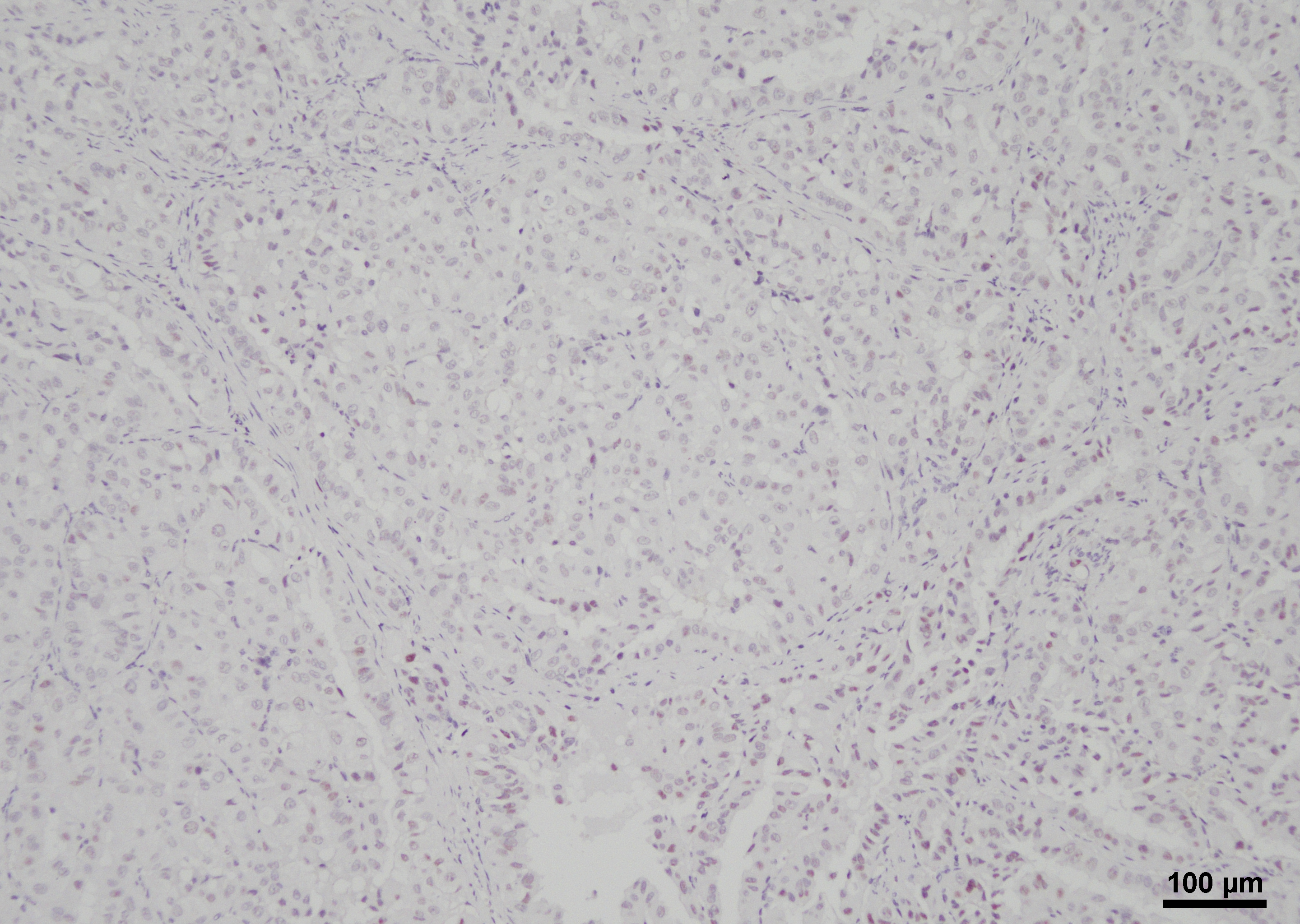

Supplement: Supplementary file 8 — Source data Fig. 5 [file 44321_2024_102_MOESM8_ESM.zip › Figure 5/5D/LDC-p-AURKA-10X.tif]

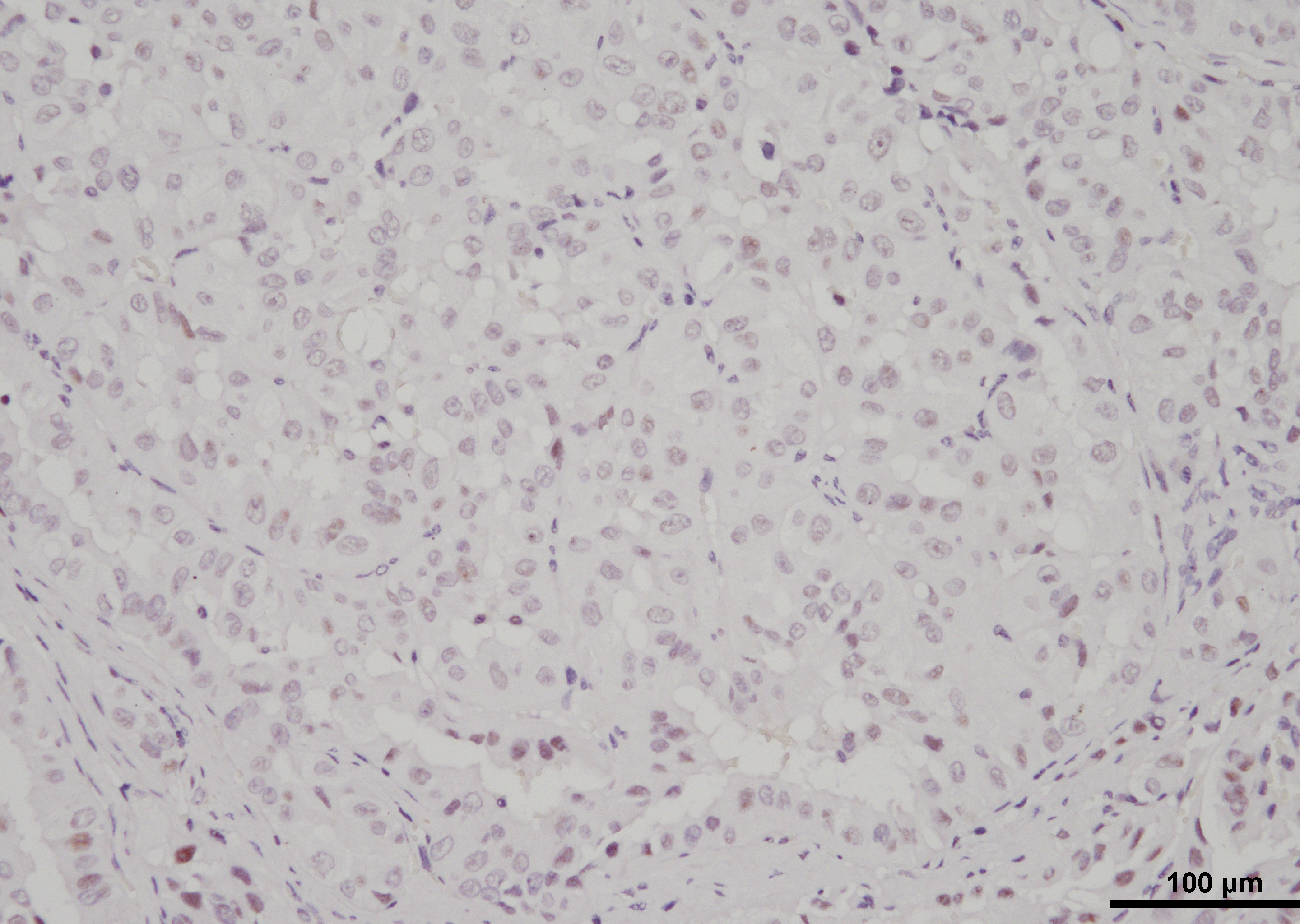

Supplement: Supplementary file 8 — Source data Fig. 5 [file 44321_2024_102_MOESM8_ESM.zip › Figure 5/5D/LDC-p-AURKA-20X.tif]

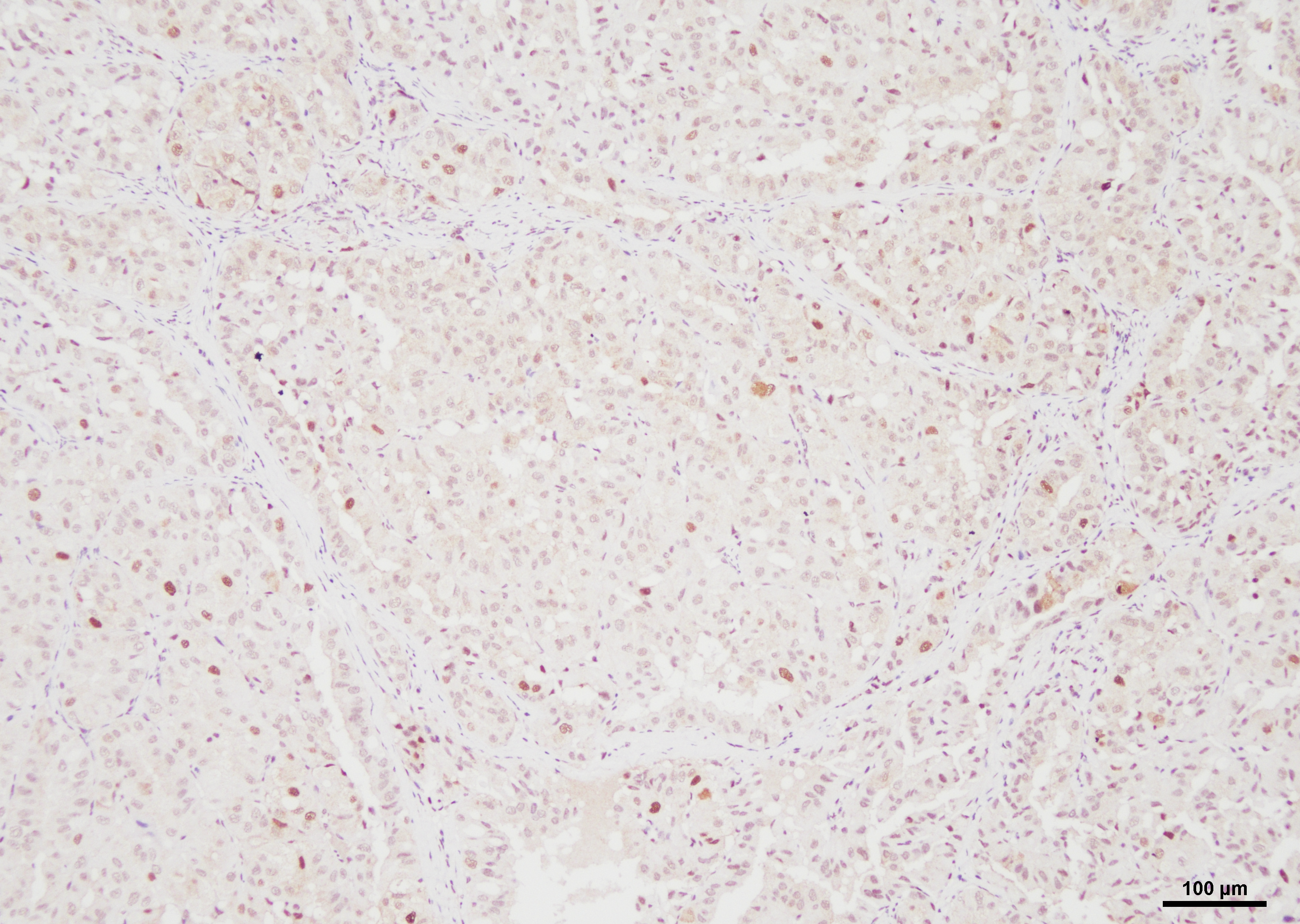

Supplement: Supplementary file 8 — Source data Fig. 5 [file 44321_2024_102_MOESM8_ESM.zip › Figure 5/5D/LDC-p-RB1-10X.tif]

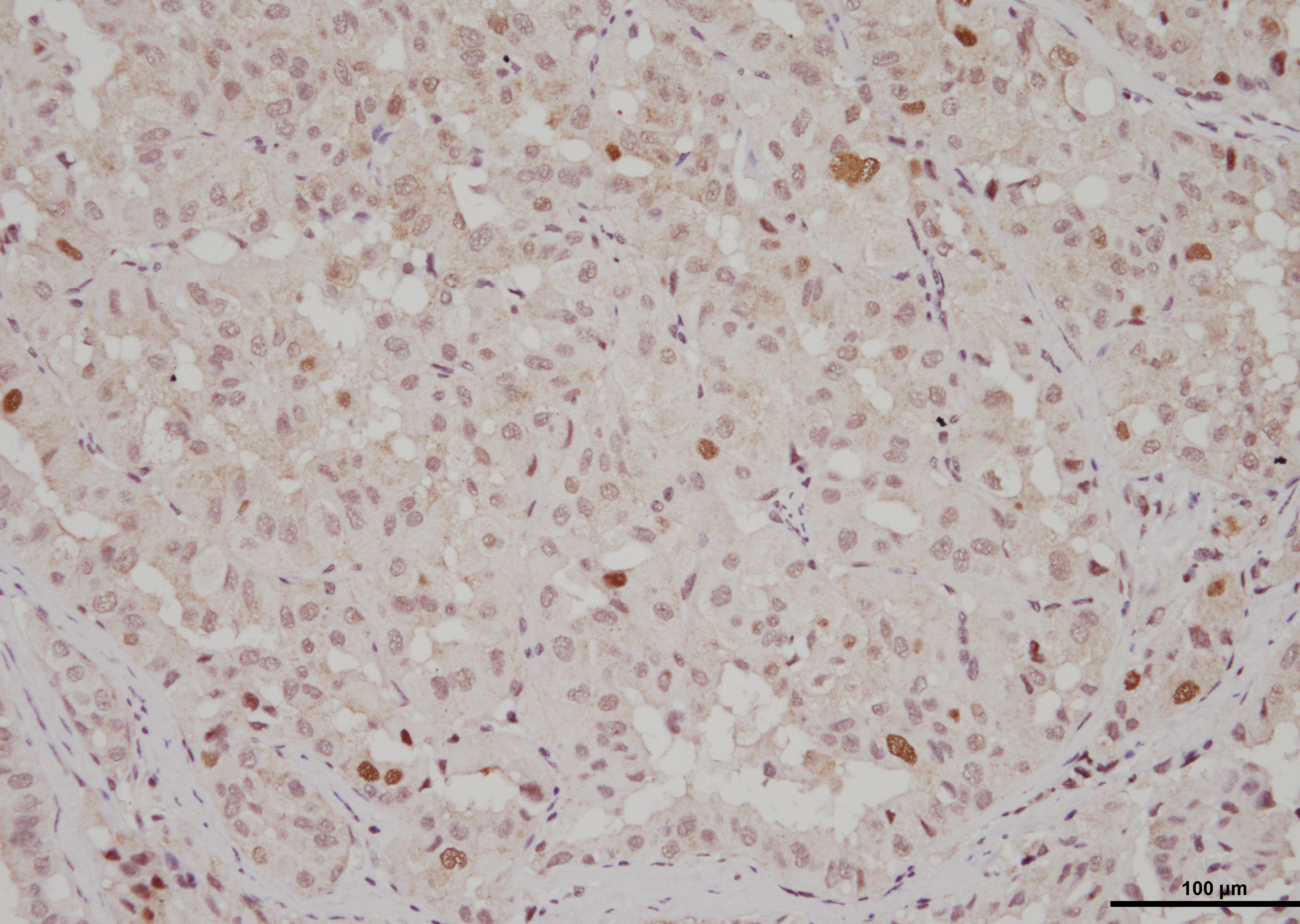

Supplement: Supplementary file 8 — Source data Fig. 5 [file 44321_2024_102_MOESM8_ESM.zip › Figure 5/5D/LDC-p-RB1-20X.tif]

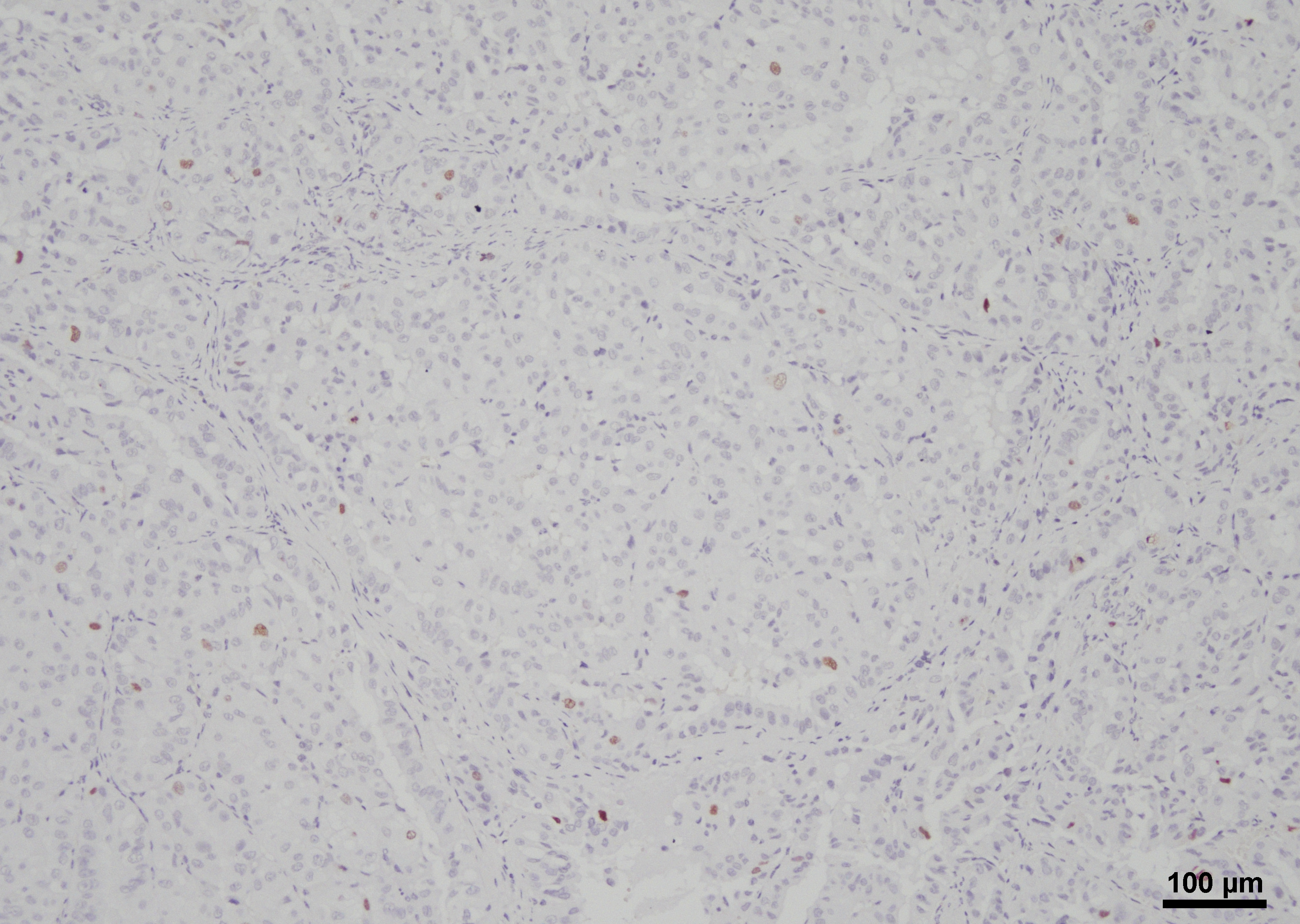

Supplement: Supplementary file 8 — Source data Fig. 5 [file 44321_2024_102_MOESM8_ESM.zip › Figure 5/5D/LDC-TPX2-10X.tif]

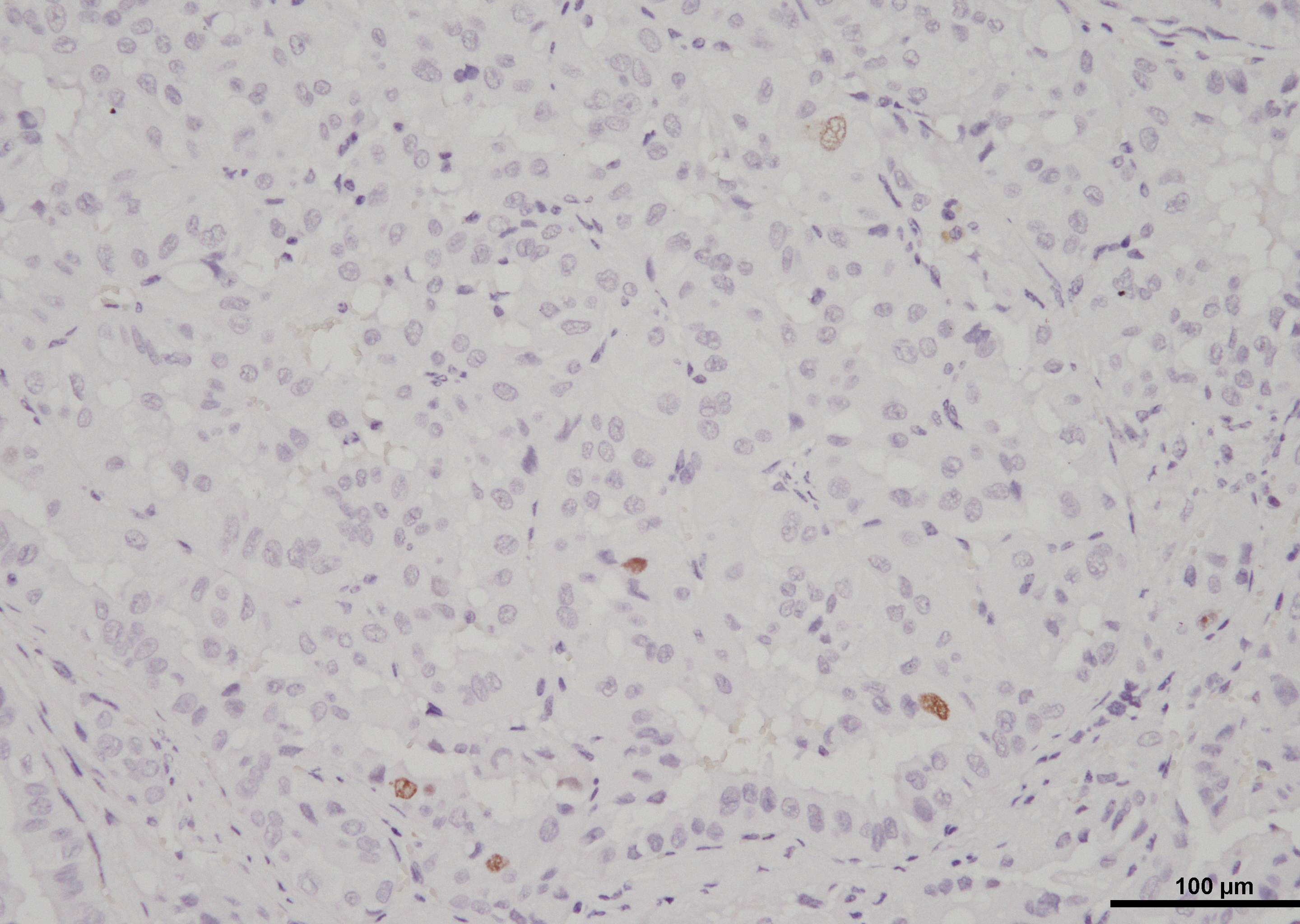

Supplement: Supplementary file 8 — Source data Fig. 5 [file 44321_2024_102_MOESM8_ESM.zip › Figure 5/5D/LDC-TPX2-20X.tif]

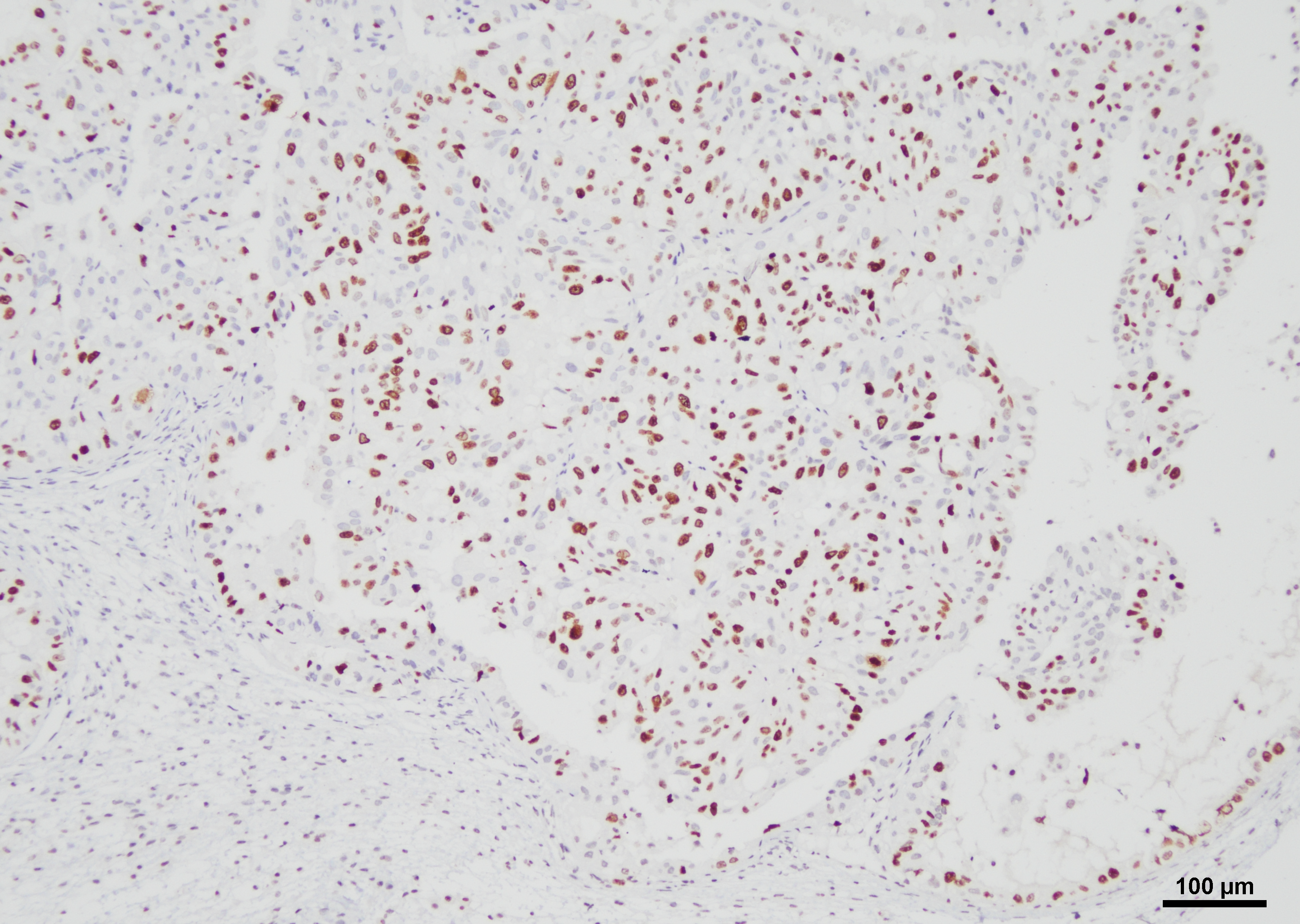

Supplement: Supplementary file 8 — Source data Fig. 5 [file 44321_2024_102_MOESM8_ESM.zip › Figure 5/5D/Vehicle-Ki67-10X.tif]

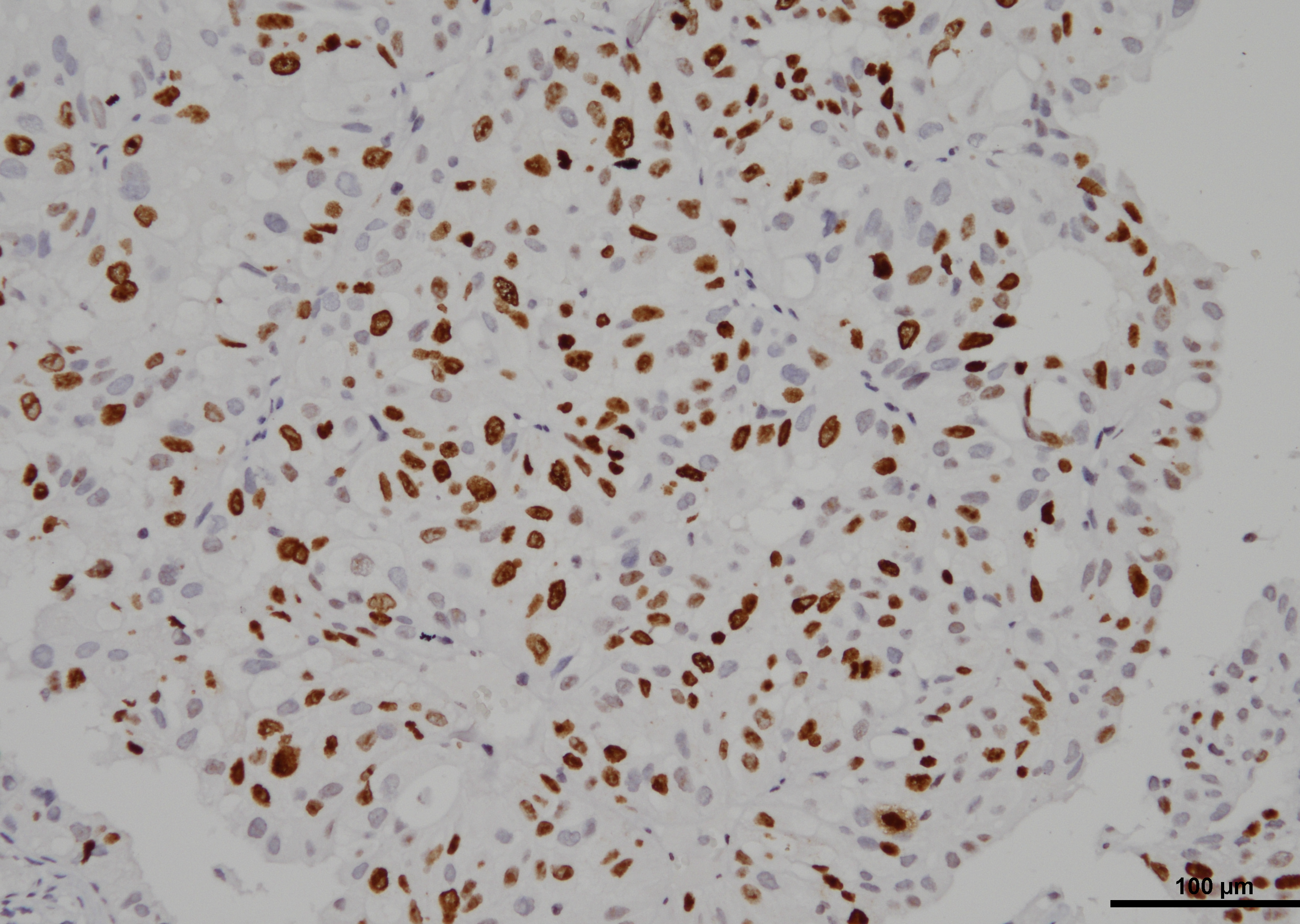

Supplement: Supplementary file 8 — Source data Fig. 5 [file 44321_2024_102_MOESM8_ESM.zip › Figure 5/5D/Vehicle-Ki67-20X.tif]

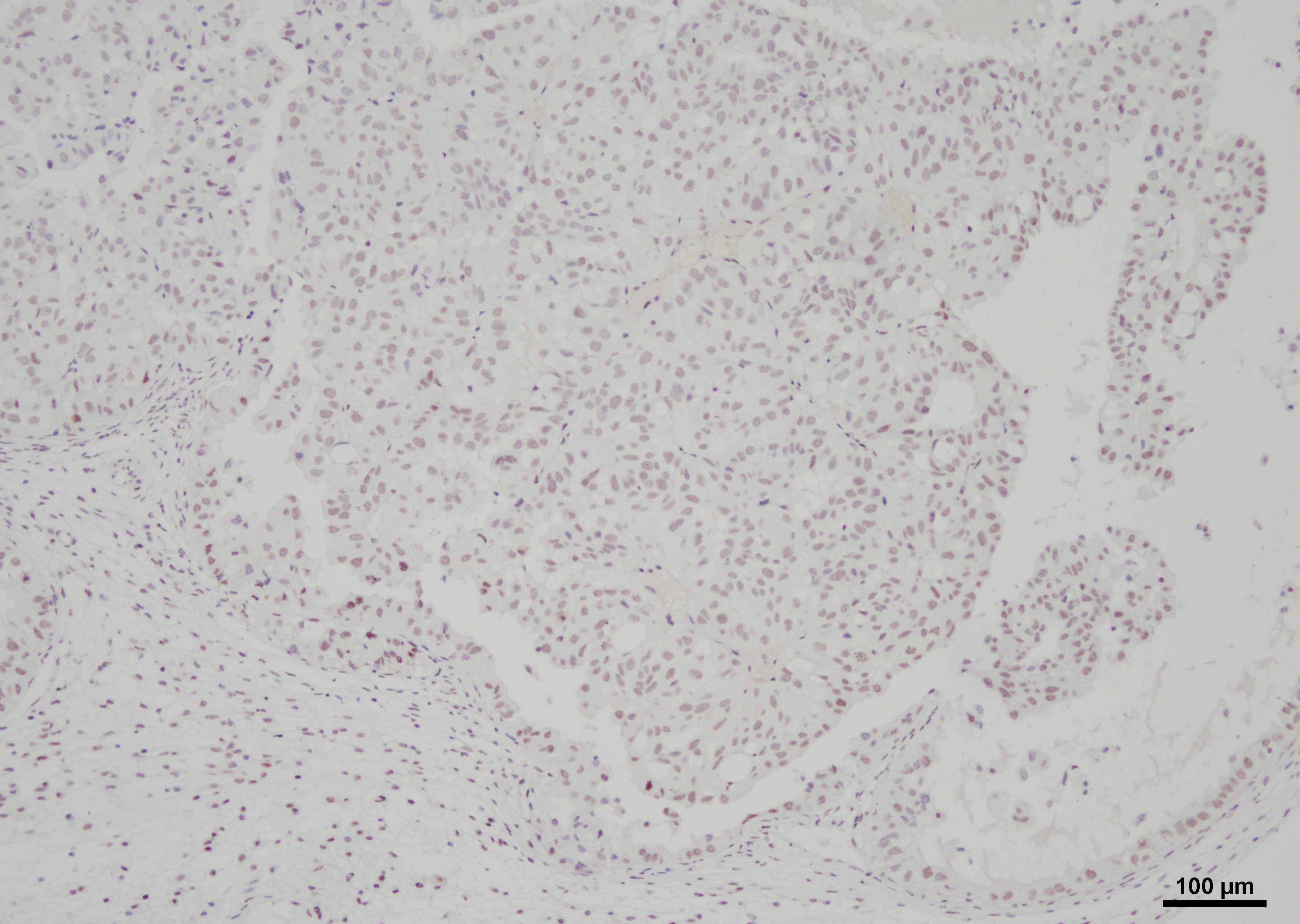

Supplement: Supplementary file 8 — Source data Fig. 5 [file 44321_2024_102_MOESM8_ESM.zip › Figure 5/5D/Vehicle-p-AURKA-10X.tif]

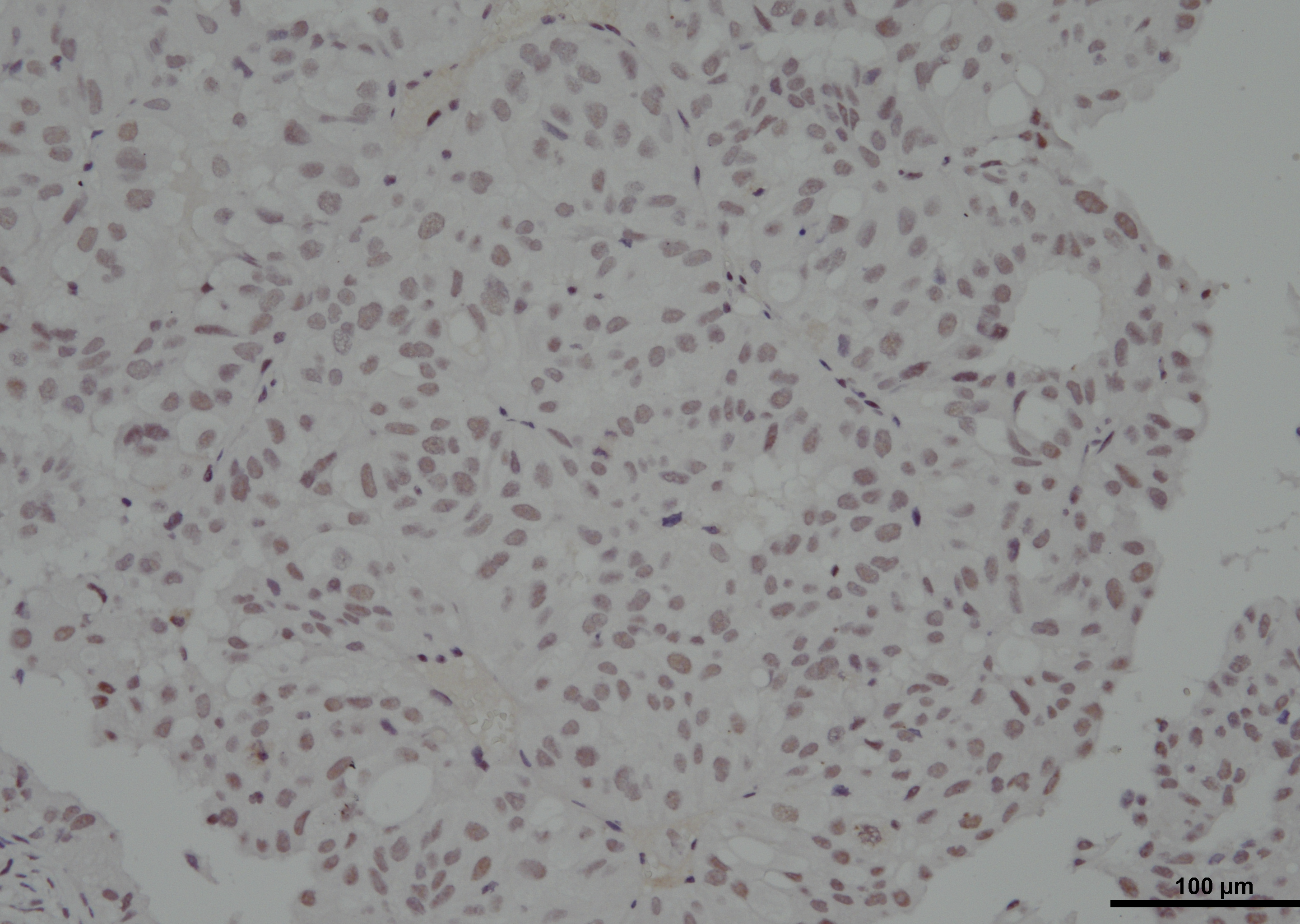

Supplement: Supplementary file 8 — Source data Fig. 5 [file 44321_2024_102_MOESM8_ESM.zip › Figure 5/5D/Vehicle-p-AURKA-20X.tif]

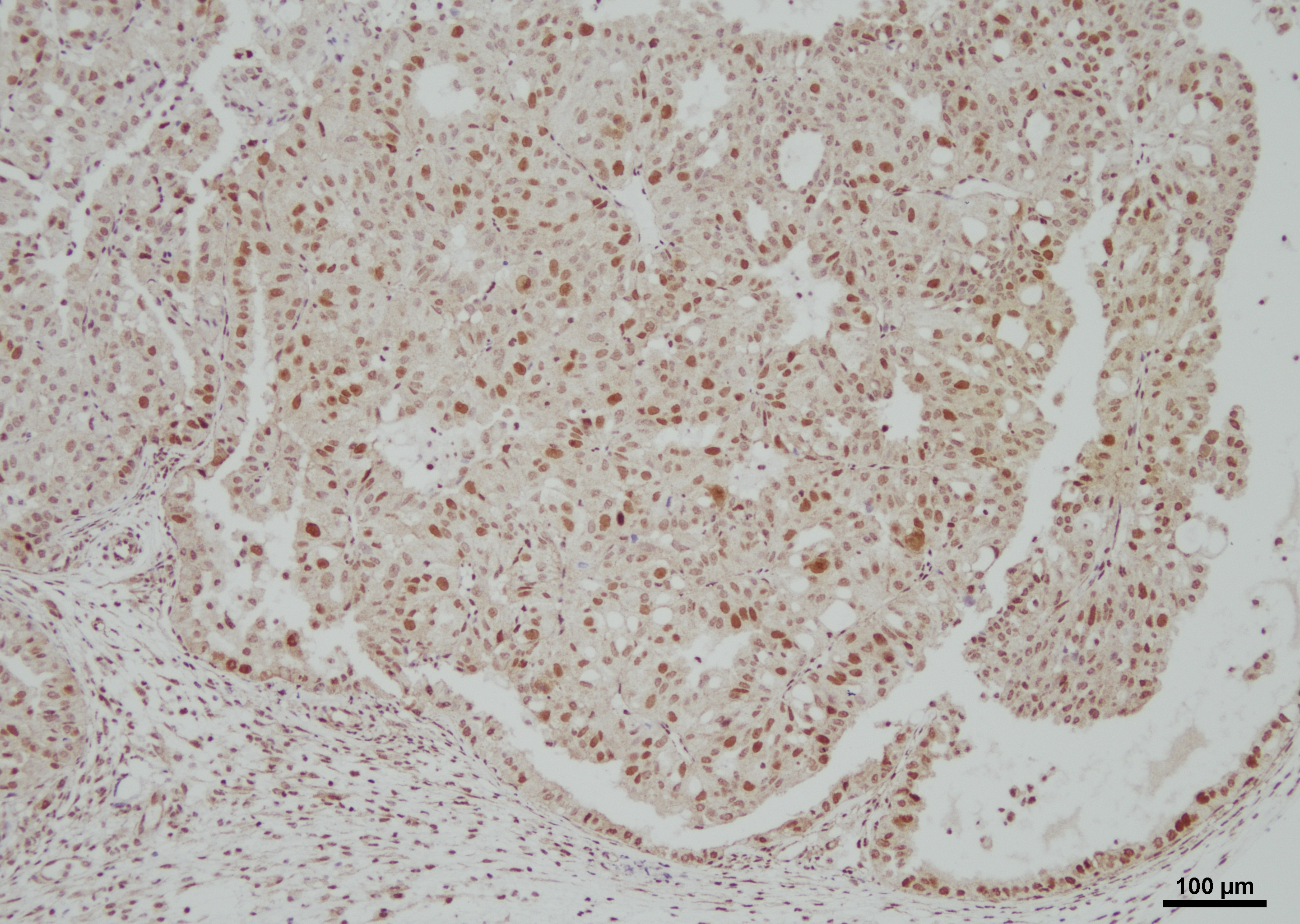

Supplement: Supplementary file 8 — Source data Fig. 5 [file 44321_2024_102_MOESM8_ESM.zip › Figure 5/5D/Vehicle-p-RB1-10X.tif]

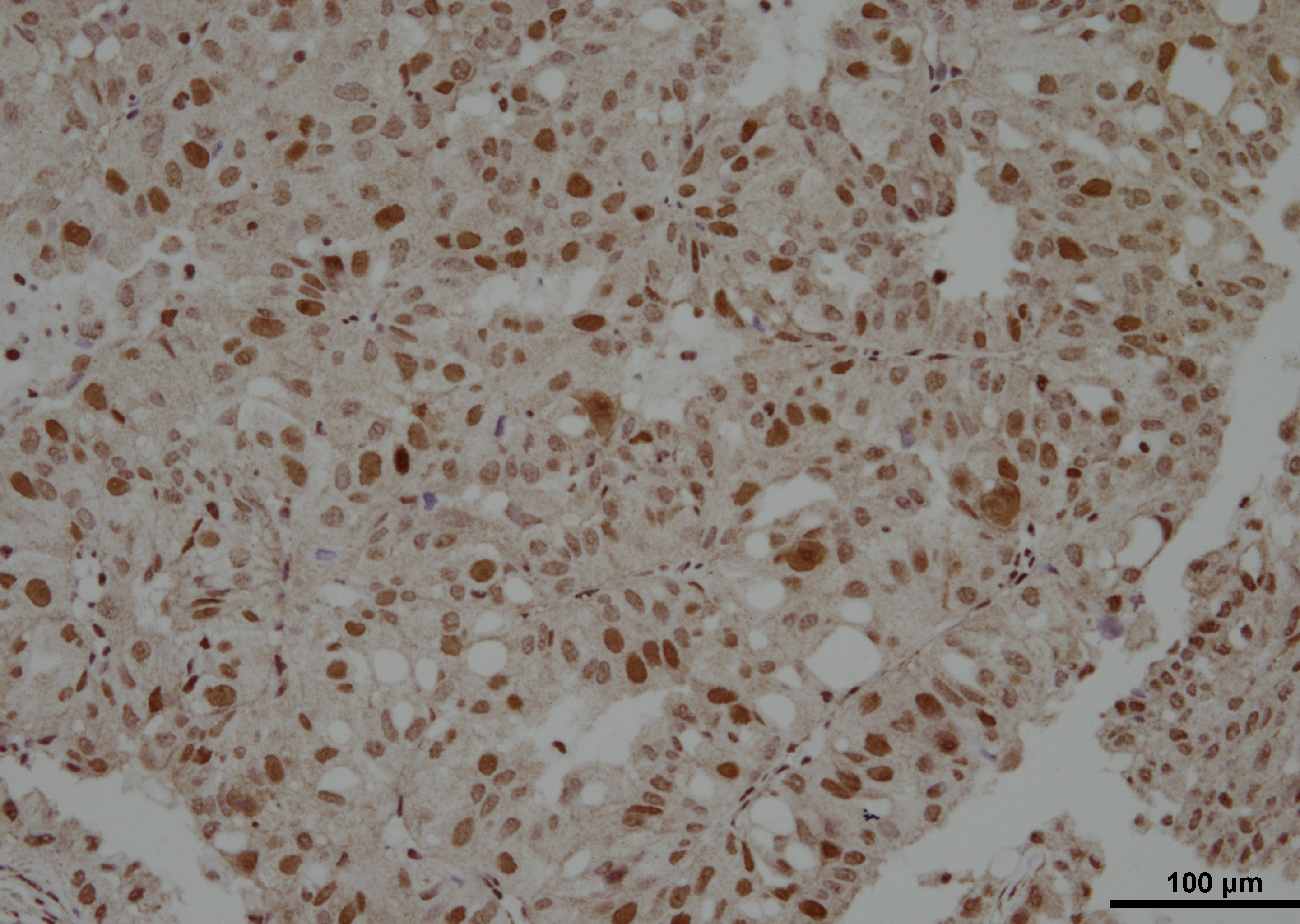

Supplement: Supplementary file 8 — Source data Fig. 5 [file 44321_2024_102_MOESM8_ESM.zip › Figure 5/5D/Vehicle-p-RB1-20X.tif]

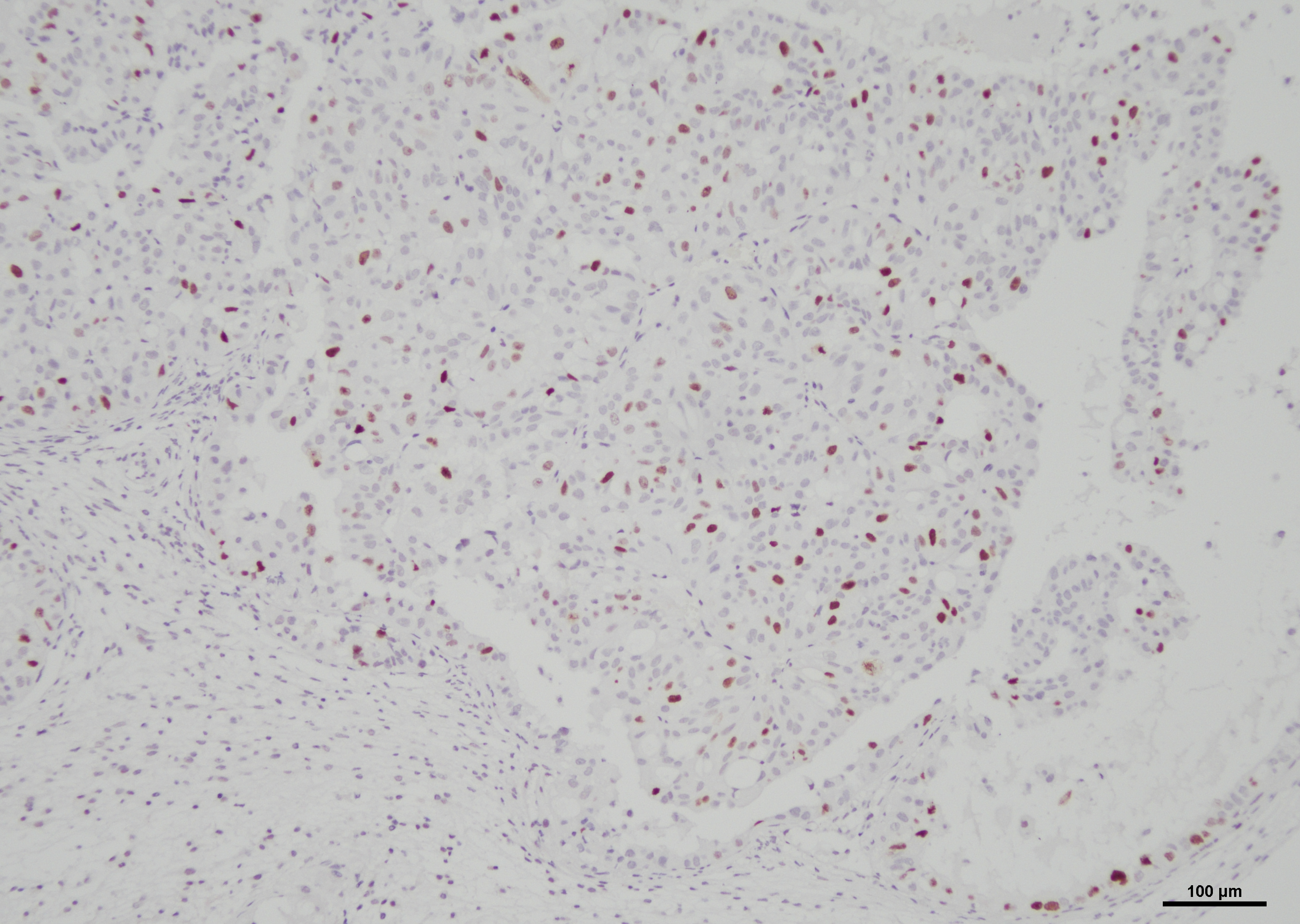

Supplement: Supplementary file 8 — Source data Fig. 5 [file 44321_2024_102_MOESM8_ESM.zip › Figure 5/5D/Vehicle-TPX2-10X.tif]

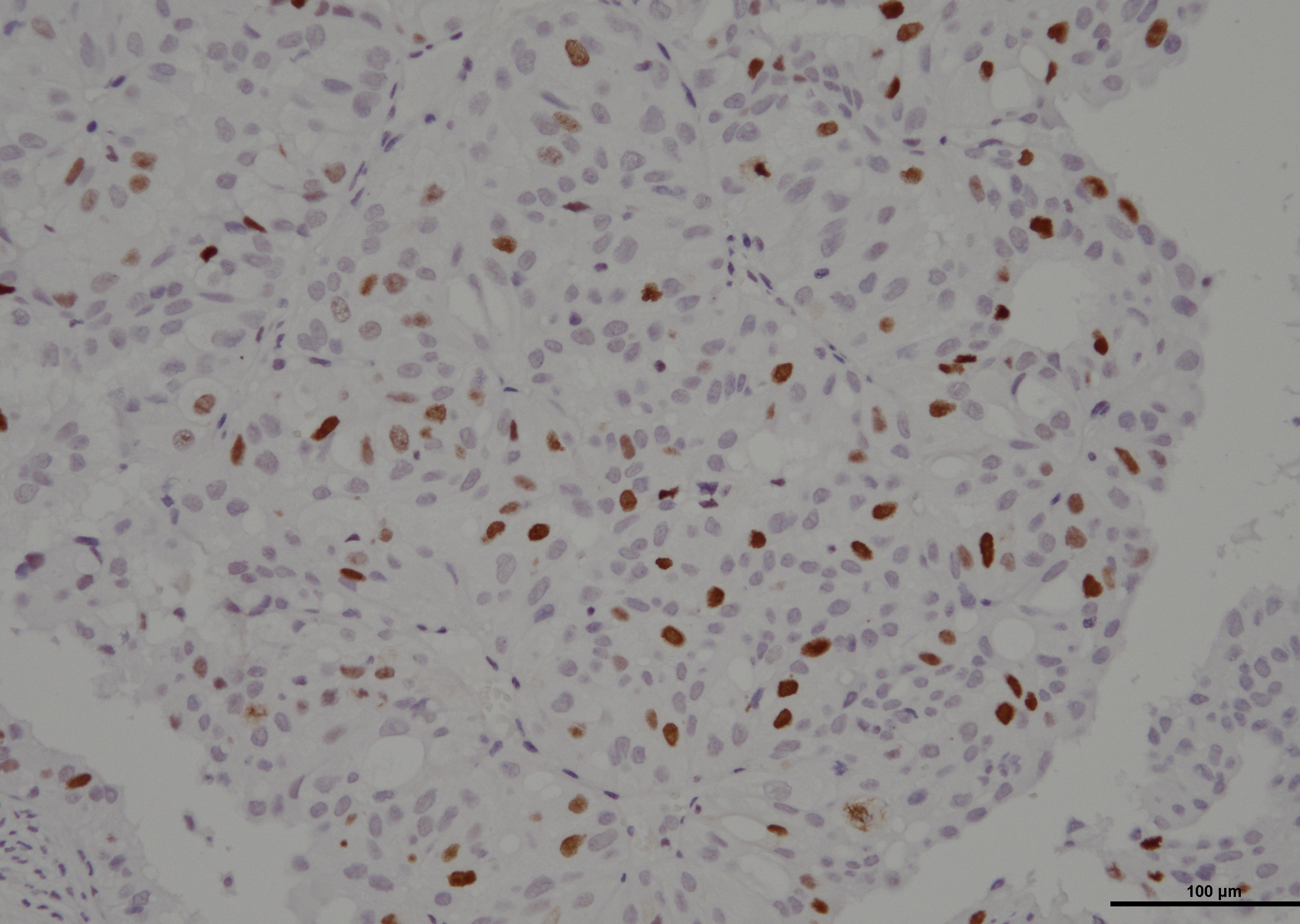

Supplement: Supplementary file 8 — Source data Fig. 5 [file 44321_2024_102_MOESM8_ESM.zip › Figure 5/5D/Vehicle-TPX2-20X.tif]
